# Farnesol and geranylgeraniol in plant reproduction: insights from Arabidopsis and beyond

**DOI:** 10.1093/jxb/erag069

**Published:** 2026-02-11

**Authors:** Małgorzata Gutkowska, Ewa Swiezewska, Joanna Rojek

**Affiliations:** Department of Biochemistry and Microbiology, Institute of Biology, Warsaw University of Life Sciences, ul. Nowoursynowska 159 blg.37, 02-776, Warsaw, Poland; Institute of Biochemistry and Biophysics PAS, ul. Pawińskiego 5a, 02-106, Warsaw, Poland; Department of Plant Experimental Biology and Biotechnology, Faculty of Biology, University of Gdańsk, ul. Wita Stwosza 59, 80-308, Gdańsk, Poland; Nanyang Technological University, Singapore

**Keywords:** Arabidopsis, farnesol, geranylgeraniol, mevalonic acid (MVA) pathway, protein prenylation

## Abstract

Isoprenoids (also called terpenoids) are a large group of natural chemical compounds. Some isoprenoids are specialized metabolites that give smell and taste to plants and provide protection against herbivores and pathogens. Production of these particular substances is specific to certain species and plant families, and is classified as secondary metabolism. In addition, numerous isoprenoids perform essential cellular functions: for example, chloroplast isoprenoids give rise to photosynthetic pigments, electron transporters, and membrane modifiers in the thylakoid membrane to adjust the correct level of photosynthetic performance and prevent oxidative damage in the chloroplasts. Similarly, some cytoplasmic isoprenoids serve a key role in the primary cell metabolism of all eukaryotic cells, forming membrane microdomains (sterols), serving as lipid anchors for prenylated proteins (geranylgeranyl and farnesyl groups), and as co-factors of protein glycosylation (dolichols). The non-steroid isoprenoids (prenyl groups of proteins and ubiquinone, dolichols) and their role in the plants are far less described than sterols. In this review, we present a summary of the knowledge on protein prenylation, but also farnesol and geranylgeraniol turnover in the cytoplasm in the context of membrane structure, biochemistry, plant physiology, and development in Arabidopsis and other plant species.

## Teaser

The lesser known cytoplasmic isoprenoids, short-chain prenyl diphosphates and prenyl groups of proteins, are reviewed with a focus on their role in plant reproduction.

## Introduction

**Fig. 1.**
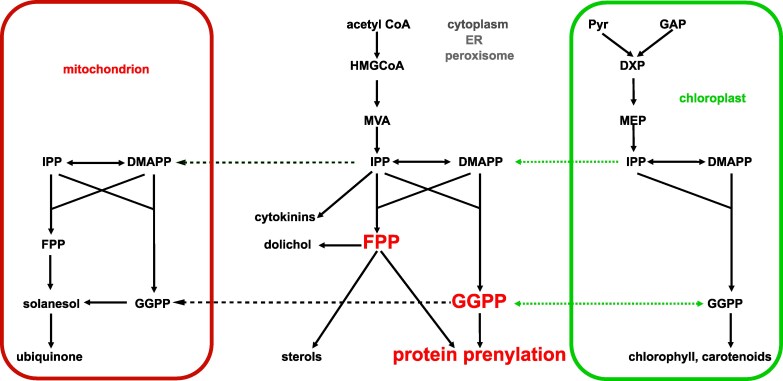
Schematic representation of isoprenoid metabolism in a plant cell. Main isoprenoid biosynthesizing pathways in chloroplast, mitochondria and cytoplasm/ER/peroxisome are depicted, with the names of central metabolites. Solid arrows show the well-established metabolic routes, dotted arrows depict the controversial steps. Abbreviations: DMAPP, dimethylallyl diphosphate; DXP, 1-deoxy-D-xylulose 5-phosphate; FPP, farnesyl diphosphate; GAP, glyceraldehyde 3-phosphate; GGPP, geranylgeranyl diphosphate; HMG-CoA, 3-hydroxy-3-methyl-glutaryl-co-enzyme A; MEP, 2-C-methyl-D-erithritol 4-phosphate; MVA, mevalonic acid; PP, isopentenyl diphosphate; Pyr, pyruvate. Green shape on the right site represents plastid and red shape on the left site represents mitochondrion. Processes being the subject of the review are highlighted in red and larger font.

Isoprenoids are lipid molecules present in all living organisms, however their amount is usually low compared with fatty acids and their derivatives. Linear isoprenoid lipids consist of a branched hydrocarbon chain and a polar head group. In eukaryotes linear isoprenoid lipids or their derivatives, reside in all compartments inside the cell, including in plastids and mitochondria. In plant cells all isoprenoids are derived from isopentenyl diphosphate (IPP) and its isomer, dimethylallyl diphosphate (DMAPP) molecules, originating from two different pools: cytoplasmic/peroxisomal and plastid (Fig. 1). The biosynthetic origins of these two pools are entirely different: the mevalonic acid (MVA) pathway starting from acetyl-CoA, and methyl erythritol phosphate (MEP) pathway starting from phosphorylated carbohydrates- photosynthetic intermediates (Hemmerlin *et al*., 2012; Pokhilko *et al*., 2015; Rodriguez-Concepcion and Boronat, 2015; Pu *et al*., 2021; Bergman *et al*., 2024). To what extent the exchange of phosphorylated sugars, and their downstream derivatives, is possible between cytoplasm and chloroplast stroma of plants remains a subject of long-lasting debate (Rodriguez-Concepcion *et al*., 2004; Skorupinska-Tudek *et al*., 2008; Huchelmann *et al*., 2016; Jozwiak *et al*., 2017; Lipko *et al*., 2023). In some experimental models, the bidirectional exchange of isoprenoid precursors/metabolites between the cytoplasm and chloroplast seems relatively active (Skorupinska-Tudek *et al*., 2008; Huchelmann *et al*., 2016; Jozwiak *et al*., 2017; Chevalier *et al*., 2024). In late-stage embryos and young seedlings, the transport of isoprenoid precursors from cytoplasm to chloroplast has been shown (Rodriguez-Concepcion *et al*., 2004; Vranova *et al*., 2019). In intact mature plants, the transport of isoprenoid precursors seems limited and unidirectional—from the chloroplast towards the cytoplasm (Lipko *et al*., 2023). Until now, the identity of the metabolites exchanged, or the mechanism of the transport remains unknown, but it must be kept in mind that in plants, the isoprenoid metabolism is more complicated than in other organisms. Generally, it is accepted that photosynthetic pigments, phytol, long-chain linear polyprenyl alcohols, plastoquinone and tocopherol side chains are MEP-derived, while sterols, ubiquinone side chain, and farnesol are MVA-derived, while dolichols are of mixed origin. The source of cytoplasmic geranylgeraniol pool is still controversial (Fig. 1).

Isoprenoids are no doubt essential across all stages of plant reproduction, especially if we consider hormonal control of the generative stage of the development, i.e. flower formation, gametogenesis, pollination, seed formation and seed dispersion. Several plant hormones are isoprenoids (Pulido *et al*., 2012). Gibberellins (diterpenoid acids, derivative of MEP pathway) promote the transition from vegetative to reproductive development, and subsequent development of both male and female floral organs (Plackett and Wilson, 2017). Abscisic acid (isoprenoid in the plastid MEP pathway) is known to control seed maturity, dormancy and germination (Ali *et al*., 2022). Cytokinin (derived from isoprenoid precursors from MVA pathway) is required for proper ovule development, female gametophyte identity (together with auxin), as well as seed and fruit development (Terceros *et al*., 2020). Brassinosteroids (steroid isoprenoids derived from MVA pathway) regulate many aspects of reproduction, from ovule and pollen formation to seed and fruit development (Lima and Figueiredo, 2024).

In a wider aspect, isoprenoids (both cytoplasmic and plastidial) can act as floral volatiles driving pollinator attraction (especially monoterpenes and sesquiterpenes, Qiao *et al*., 2021) and reproductive fitness (carotenoids in flower/fruit pigmentation, Watkins, 2023). Nevertheless, in this review we focus on the developmental aspects of short-chain isoprenoid biology, apart from the hormonal and ecological roles the derivatives of farnesol and geranylgeraniol play in plant life.

In this article, we review the biochemistry of short prenyl alcohol biosynthesis and degradation. We also focus on the role of FPP, GGPP and their downstream metabolites on plant gametophytes and seed development. This new perspective exemplifies how basic metabolism influences processes at the tissue/organ/organism levels. It also helps us pose questions concerning the mechanisms of prioritization of certain metabolic processes that share common precursor pools and interwoven feedback regulation of key enzymes. Since gametophytes and young embryos are fed with metabolites by the surrounding sporophytic tissues, they appear as a good model to study inter-tissue metabolite exchange. So far, isoprenoid metabolism in plants has not been discussed in this context.

## Transport of isoprenoid metabolites from the sporophyte to gametophytes and young embryo

**Fig. 2.**
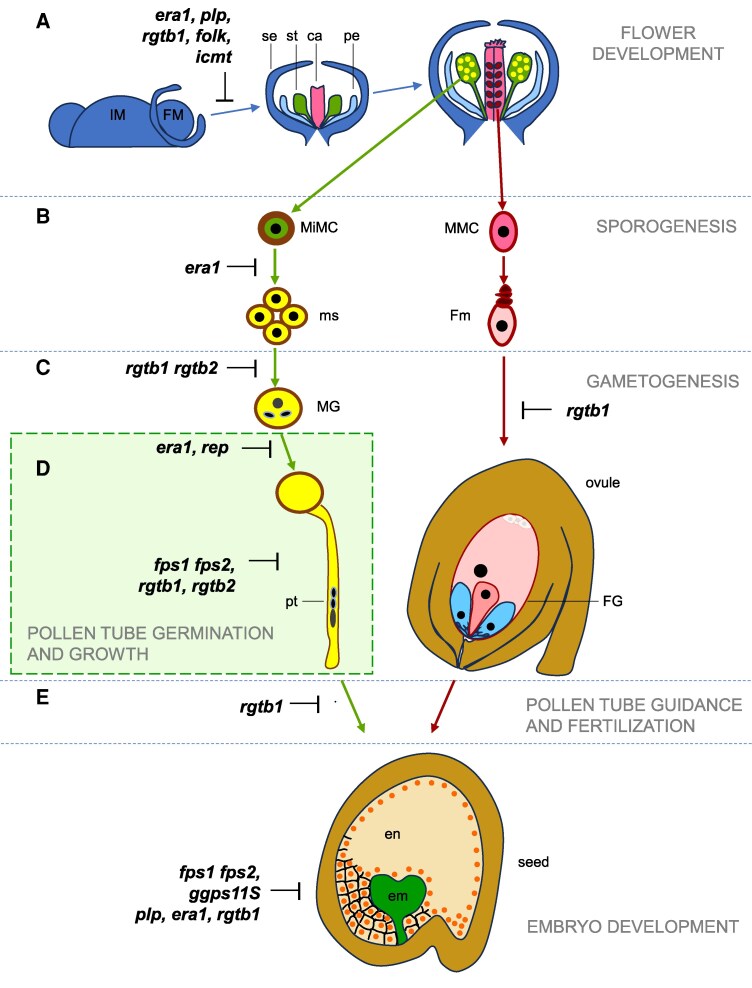
Schematic representation of influence of the failure of isoprenoid-dependent processes on angiosperm generative development. Stages at which development of knock-out mutants in isoprenoid biosynthesis genes stops are marked on the scheme. Blue arrows—flower development; green arrows on the left site—male gametophyte development; red arrows on the right site—female gametophyte development. (A) The floral meristem differentiates at the merge of the inflorescence meristem. Inside the flower, four whorls of organs differentiate: sepals, petals, stamens, and carpels. Upper parts of stamens form anthers, and carpels fuse to form pistils, inside which ovules carrying female gametophytes develop. Arabidopsis mutants in protein farnesylation and farnesol recycling, as well as in Rab protein geranylgeranylation, show pronounced disturbance in flower development and flower organ identity. (B) Meiosis inside anther tissue leads to the formation of a tetrad of haploid microspores. Meiosis inside the tissue of the ovule and further degeneration of three post-meiotic cells lead to the formation of one functional haploid megaspore. (C) Further mitotic divisions inside each of the microspores produce three-cellular male gametophytes containing two sperm cells surrounded by a vegetative cell cytoplasm. Three consecutive mitoses inside the megaspore lead to the formation of a mature female gametophyte containing one egg cell and one central cell. Arabidopsis mutants in protein prenylation are affected in the gametophytes formation. (D) The vegetative cell of pollen germinates on a stigma and forms a protrusion called a pollen tube. The sperm cells migrate inside the pollen tube towards the female gametophyte. Arabidopsis mutants in protein prenylation and farnesyl diphosphate synthesis are affected in the pollen tube-ovule recognition. (E) The pollen tube guided by the female gametophyte-released signals reaches the ovule entrance and releases the sperm cells inside the female gametophyte. One sperm cell fertilizes the egg cell, second sperm cell fertilizes the central cell. (F) The fertilized egg cell develops into an embryo, and the fertilized central cell develops into nourishing tissue called endosperm. Arabidopsis mutants in protein farnesylation, farnesyl diphosphate, and geranylgeranyl diphosphate synthesis show defective embryo development.

To better understand the influence of the inhibition of the MVA-derived isoprenoid synthesis on the plant lifecycle, some basic facts on the development of the gametes and the seed should be recalled (summarized in a graphical form on Fig. 2). In Angiosperms, developing gametophytes are nourished by maternal tissues. After microsporogenesis, the developing male gametophyte is dependent on substances provided by the sporophytic nourishing layer of the anther called tapetum (Moreira *et al*., 2025; Zhou and Dobritsa, 2025). After successful pollination, the mobilization of pollen grain reserves, and the ability to use resources deposited by the sporophytic transmitting tract of the flower, determine pollen fitness and influence pollen fertilization success (Hafidh and Honys, 2021). On the female side, a structure called funiculus connects the ovule to the sporophytic maternal tissue called gynoecium. The funiculus is therefore a key route for transporting nutrients, minerals, sugars and maternal signals to the ovule and then to the developing embryo and seed (Larsson *et al*., 2017; Rojek *et al*., 2021a; Rojek *et al*., 2021b; Ferreira *et al*., 2024). In the ovule, double fertilization of the egg cell and the central cell by two sperm cells of the pollen produces the embryo and the nutritive tissue—the endosperm. Both products are completely enclosed within the ovule, and all maternally provided resources must be transferred to the offspring through the ovule vasculature, mainly the phloem. At the maternal-filial boundary the resources and developmental signals are passed on to the endosperm and embryo through a process called phloem unloading (Povilus and Gehring, 2022; Borghi, 2025). The endosperm inside the forming seed is essential for proper embryo development and seed viability. During the early stages of seed development (until the globular stage), the majority of nutrients and growth regulators are delivered to the embryo via the suspensor, which connects the embryo proper to the surrounding maternal tissues and the endosperm (Lafon-Placette and Köhler, 2014). In plants like Arabidopsis, the second pathway of nutrient uptake to the embryo involves transport through the endosperm, and this route dominates at the later stages of embryo development, i.e. from the early heart stage onwards, and is concomitant with the degeneration of the suspensor cells and endosperm cellularization (Doll and Ingram, 2022).

A question remains as to how and to what extent maternal sporophytic tissues can feed the developing gametophytes and young developing embryos inside the seed with isoprenoid lipids or their precursors. The issue of whether the isoprenoids are transported to the gametophytes and the embryo can be answered by analysis of the mutant series in *Arabidopsis thaliana.* Gametes of Arabidopsis mutants with main MEP pathway gene knock-outs are fertile, meaning that the MVA pathway serves as the main source of isoprenoids for developing gametophytes (Xing *et al*., 2010; Vranova *et al*., 2013; Gupta and Hirschberg, 2022; Basallo *et al*., 2023). Arabidopsis knock-out mutants in genes from the MVA pathway are male sterile due to maternal sporophytic defects on the developing pollen grains, i.e. lack of certain isoprenoid metabolites in the sporophytic tapetum layer of the anther (Okada *et al*., 2008; Suzuki *et al*., 2009; Ishiguro *et al*., 2010; Kobayashi *et al*., 2018). The best described case is mutation in the genes encoding a regulatory enzyme 3-hydroxy-3-methyl-glutaryl-CoA reductase (HMGR). HMGR in Arabidopsis is encoded by two genes: more ubiquitous *HMGR1* and *HMGR2* (Suzuki *et al*., 2004). In this plant *HMGR* transcription is very high in mature anthers and mature pollen (https://bar.utoronto.ca/). It is worth mentioning that in flowers at developmental stage 11/12, when the male gametophyte undergoes second mitotic division and the nourishing tapetum layer of an anther starts degenerating, both *HMGR1* and *HMGR2* genes reach high level of transcription (https://bar.utoronto.ca/). These findings are in line with the analysis of *HMGR1* promotor activity in GUS assays and the observed *hmgr1* phenotype (Suzuki *et al*., 2004). *HMGR1* is highly expressed in the anthers and pollen of immature flowers, and *HMGR2* in pollen only, as was experimentally observed by *in situ* RNA hybridization (Suzuki *et al*., 2009). In the *hmgr1* mutant the pollen produced is unable to germinate on the wild type stigma, while the pollen derived from heterozygous *HMGR1/hmgr1* plants is fertile. These observations suggest that the tapetum is a source of mevalonate derivative, crucial for pollen development. The authors showed that the compound produced in the tapetum and transported to pollen grains, that reverses the male infertility phenotype of *hmgr1* mutant, is a product of farnesyl diphosphate catalyzed condensation, squalene, an early non-cyclic sterol derivative (Suzuki *et al*., 2004). Total lack of HMGR activity due to double mutation in *HMGR1* and *HMGR2* is pollen-lethal, which is in line with the transcription data—both genes are highly expressed inside developing pollen (Suzuki *et al*., 2009). The reason for pollen lethality was determined to be endoplasmic reticulum hypotrophy and failure in normal development of the intracellular membranes, probably due to lack of functional sterols. Furthermore, mutant plants homozygous for protein prenyltransferases, or sterol biosynthesis mutants, show maternal sporophytic defects of pollen development (*rgtb1*–Rojek *et al*., 2021b; *era1*–Verges *et al*., 2021; *smt1*- Diener *et al*., 2000).

Less is known about the maternal sporophytic defect of female gametophyte development, but low efficiency in mature ovule formation in some homozygous mutants have been described (*rgtb1*–Rojek *et al*., 2021a; *era1*–Verges *et al*., 2021; *smt1*–Diener *et al*., 2000). In all these cases, the decreased (bidirectional) transport through the funiculus, or perturbed sieve element development in the funiculus affecting its functionality, might be the reasons for female gametophyte failure. In case of *HMGR* genes, the female gametophyte produced on *HMGR1/hmgr1 hmgr2/hmgr2* plant was fertile, unlike the male one (Suzuki *et al*., 2009), also suggesting possible transport of mevalonate derivative inside the developing female gametophytes (otherwise half of the ovules carrying the *hmgr1 hmgr2* genotype would be unable to develop due to gametophytic failure).

The maternal effect of the sporophyte on embryo development is obvious in many embryo-lethal mutants in the genes encoding enzymes involved in non-sterol and sterol isoprenoid biosynthesis. The young developing embryo, hidden well inside the maternal tissues, relies on the maternal supply of isoprenoid precursors (Gao *et al*., 2019; Vranova *et al*., 2019). The stage at which the embryo halts in development occurs simultaneous to a major decrease in symplastic transport of nutrients through the phloem of the ovule funiculus, i.e. octant/early globular stage (*fps1 fps2*- Closa *et al*., 2010; Robert, 2019; Ferreira *et al*., 2024). Some mutants in the isoprenoid biosynthesis pathway in the cytoplasm halt embryo development after programmed cell death of the suspensor, when the main transport route of sugar and other metabolites changes from a symplastic suspensor-dependent route to an endosperm-dependent apoplastic route (Lafon-Placette and Köhler, 2014), i.e. at the transition from globular to early heart stage (e.g. many sterol biosynthesis mutants: *smo-1*, Song *et al*., 2019; *smo-2*, Zhang *et al*., 2016; *hyd-1*, Pullen *et al*., 2010, and ubiquinone biosynthesis mutants: *ppt*, Okada *et al*., 2004, and geranylgeranyl diphosphate synthase mutant *ggps11-4*, Ruiz-Sola *et al*., 2016a).

The molecular identity of the isoprenoid compounds that are transported symplastically by phloem from sporophytic tissues of the ovary to the female gametophyte, and later to the embryo is not known, but they are probably free alcohols (e.g. farnesol or geranylgeraniol) or their acyl esters (alcohol phosphates, as charged molecules seem rather unlikely). The larger and much more hydrophobic downstream products like sterols, dolichols, or ubiquinone side chain are of low solubility in the phloem sap; however, transport of some sterols in the phloem was documented by radioactive trace localization monitoring (O’Brien *et al*., 2005). The early embryonic lethality of the Arabidopsis mutants in genes encoding farnesyl diphosphate synthase and geranylgeranyl diphosphate synthase confirm the notion of short chain isoprenoids transport (Closa *et al*., 2010; Ruiz-Sola *et al*., 2016a). The pattern of expression of the corresponding genes also provides indirect proof for this assumption—farnesyl diphosphate (FPP) and geranylgeranyl diphosphate (GGPP) synthases are widely expressed in the sporophytic tissues of the ovule, but not inside the female gametophyte or young embryo (Cunillera *et al*., 2000; Keim *et al*., 2012; also analysis of the expression patterns of the FPP and GGPP synthases extracted from wide transcriptomic screens, see https://bar.utoronto.ca/; Beck *et al*., 2013). Surprisingly, starting from late globular stage, chlorophyll is synthesized inside the embryo of some plants, including Arabidopsis (Tejos *et al*., 2010), and photosynthesis becomes active inside certain layers of embryonic cells (Allorent *et al*., 2015; Sela *et al*., 2020). Later on, the chloroplasts inside the embryo lose their photosynthetic activity—the walking stick stage embryo does not contain chlorophyll (Liebers *et al*., 2017). Hence the issue of metabolic cross-talk in isoprenoid synthesis during embryo development is much more complex than in the case of gametophyte development.

### Short chain all-*trans*(E)-isoprenoids: at the cross-road of cytoplasmic isoprenoid metabolism

#### Farnesyl and geranylgeranyl diphosphates: chemical and biophysical properties

**Fig. 3.**
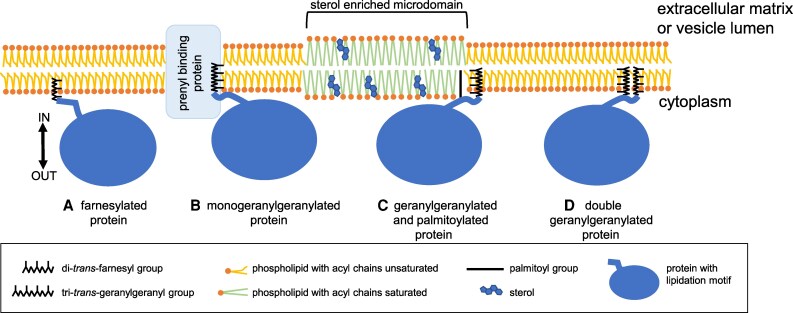
Schematic representation of prenyl groups localization in the biological membranes. Modes of prenyl group interaction with the bilayer depicted according to Atsmon-Raz and Tieleman (2017); Levental *et al*. (2010) and Kulakowski *et al*. (2018). (A) A farnesylated protein interacts with the membrane transiently with fast “on/off” rates; (B) a monogeranylgeranylated protein interacts with the membrane more stably, possibly being recognized by prenyl-interacting proteins; (C) a protein modified by both geranylgeranyl and palmitoyl group resides at the merge of the sterol enriched domain; (D) a double geranylgeranylation of Rab protein on the adjacent cysteines provides stable interaction with the membrane. Prenylated proteins are excluded from the sterol-enriched domains. The scale of the length of isoprenoid groups in comparison to fatty acid chains is preserved. Graphical symbols are described in the key.

Short-chain isoprenoid diphosphates are fairly soluble in water (FPP 0.0807 mg ml^–1^, GGPP 0.00463 mg ml^–1^; Cayman Chemical https://www.caymanchem.com/) but also, in the case of GGPP, insert spontaneously in the membrane. Branching of the isoprenoid chain in FPP and GGPP decreases the length of these molecules compared with fatty acids containing the same number of carbon atoms. The 15-carbon FPP molecule is shorter than 14-carbon myristic acid (the main carbon chain length of FPP is 12 carbons plus methyl groups branching), the geranylgeranyl group is 16-carbon long (not counting for the branching) and of the same length as the saturated palmitoyl group (Atsmon-Raz and Tieleman, 2017). The molecule length accounts for the intracellular membrane interaction. While the farnesyl hydrocarbon chain cannot reach deeply into the membrane leaflet, the geranylgeranyl group can encompass one leaflet of the membrane, as judged from molecular dynamics simulations—see Fig. 3 (Atsmon-Raz and Tieleman, 2017; Koukos *et al*., 2023). Multiple branching in the hydrocarbon chain of FPP or GGPP imposes consequences on their interactions with the acyl chains of the membrane (Fig. 3); in particular, this issue is important for prenylated proteins, as discussed in the following paragraphs (Levental *et al*., 2010; Jang *et al*., 2015). All internal double bonds in FPP and GGPP are in *trans*(E)*-*configuration, which additionally makes the conformation of the molecule rigid (Entova *et al*., 2019). To summarize, short-chain all*-trans*-isoprenoid phosphates are lipids of strikingly different biophysical properties than fatty acids or cholesterol, which may translate to their molecular functions.

### Farnesol function and biosynthesis in plants

#### Farnesyl diphosphate as a metabolic precursor in eukaryotes

Farnesyl diphosphate is produced in all eukaryotes in the cytoplasm, with plants being no exception. In all eukaryotes it is a precursor for sterols, which are the major non-acyl lipid membrane components, dolichols, side chain of ubiquinone, and lipid anchor used for modification of proteins (farnesylation) (Fig. 1). In some plant families, apart from the crucial role in primary metabolism, farnesyl diphosphate is also a precursor of sesquiterpenes and triterpenes—large and extremely diversified groups of secondary metabolites, abundant in Angiosperms and Gymnosperms (Tholl, 2006).

#### Mutants of all-trans-farnesyl diphosphate synthases

**Fig. 4.**
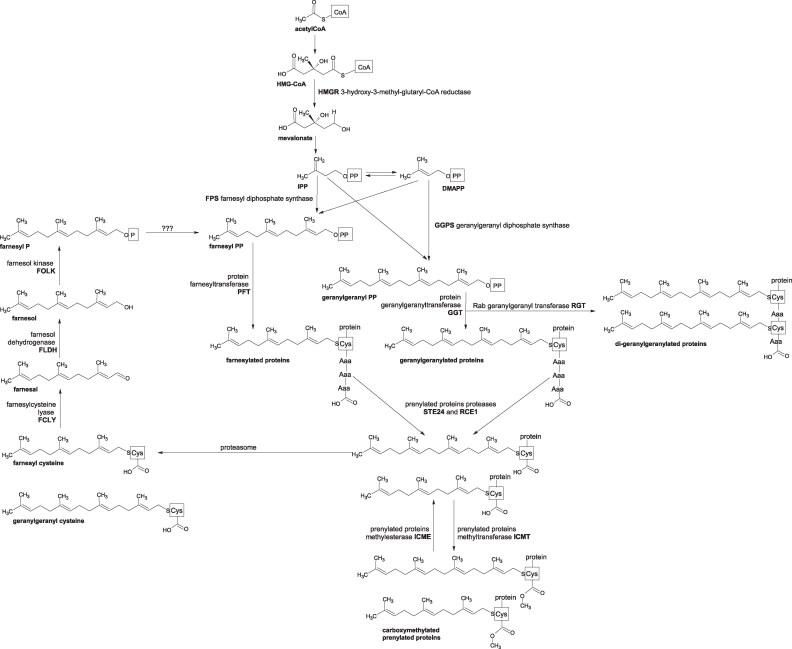
Biochemical pathways involved in farnesylated and geranylgeranylated protein synthesis and prenyl groups salvage pathway. For clarity only isoprenoids and related metabolites are shown with full chemical formulas. DMAPP, dimethylallyl diphosphate; FPP, farnesyl diphosphate; GGPP, geranylgeranyl diphosphate; HMG-CoA, 3-hydroxy-3-methyl-glutaryl-co-enzyme A; IPP, isopentenyl diphosphate. Figure prepared using ChemSketch.

In eukaryotes, cytosolic farnesyl diphosphate synthase (FPS) catalyzes the condensation of an allylic precursor DMAPP and two molecules of IPP in consecutive reactions in a head-to-tail manner, yielding the 15-carbon di-*trans*(E,E)*-*FPP (Fig. 4). In plants, farnesyl diphosphate synthases constitute small gene families, with more members in those species producing large amounts of secondary metabolite sesquiterpenes (e.g. *Asteraceae*, *Umbelliferae*, etc.—Petrova *et al*., 2024; Amen *et al*., 2025). In Arabidopsis, FPP synthase is encoded by two genes: *FPS1*—producing two isoforms: FPS1S cytosolic and FPS1L mitochondrial, and *FPS2*—producing one isoform targeted to the cytosol (Cunillera *et al*., 1996, 1997; see Table 1). The mitochondrial isoform FPS1L provides the substrate for ubiquinone side chain synthesis, while the two cytoplasmic isoforms provide precursors for sterols (and brassinosteroids) and dolichols biosynthesis, as well as for protein prenylation (Figs 1, 4). The Arabidopsis FPS1S isoform is more ubiquitously expressed in the plant sporophyte, but has lower catalytic activity (Closa *et al*., 2010), whilst FPS2 isozyme is mainly responsible for FPP synthesis in developing and germinating seeds and young seedlings, but has higher catalytic efficiency (Closa *et al*., 2010; Keim *et al*., 2012; see Table 1). The activity of FPS1S and FPS2 in the mature sporophyte of Arabidopsis is redundant, and single mutants in each of the *FPS* genes are viable and fertile (Closa *et al*., 2010). A slightly lower sterol amount is noticed compared with wild-type plants in the sporophytic tissues of mutants (Closa *et al*., 2010). In seeds, the relative content of sitosterol in the *fps2* mutant in comparison to wild type is reduced to 40% (Keim *et al*., 2012), in line with the nearly exclusive expression of *FPS2* in seeds. The *fps2* mutant shows positive feedback regulation of 3-hydroxy-3-methylglutaryl-CoA reductase (HMGR) activity (Closa *et al*., 2010).

**Table 1.** Summary of enzymatic functions and phenotypic manifestations of mutants in isoprenoid metabolism genes in Arabidopsis

Enzyme EC no	Locus in Arabidopsis	Isoform	Male gametophyte	Female gametophyte	Seed	Flower and silique	Citation
FPS EC 2.5.1.10	At5g47770	FPS1L-mitochondria	Single mutant have fertile pollen; about 2% genetic transmission of *fps1 fps2* double mutation; *fps1 fps2* pollen grains mature and germinate, pollen tube grow shorter than WT	Normal genetic transmission of single and double mutation	Normal in single mutants, more than 50% embryo abortion of *fps1 fps2,* embryo halted at octant/early globular stage	Normal in single mutants	Closa *et al*. (2010)
		FPS1S-cytosol					
	At4g17190	FPS2-cytosol					
GGPS EC 2.5.1.29	At1g49530	GGPS1- mitochondria	Normal genetic transmission	Normal genetic transmission	No data		Ruiz-Sola *et al*. (2016b)
	At2g18640	GGPS3-cytosol	Not expressed in generative organs				
	At2g23810	GGPS4-cytosol	High expression in anthers; no genetic or developmental data	No data			
	At4g36810	GGPS11S-cytosol	Normal genetic transmission	Normal genetic transmission	25% of embryos halted at transition from globular to heart stage	n.a.	Ruiz-Sola *et al*. (2016a); Ruiz-Sola *et al*. (2016b)
		GGPS11L- chloroplast					
Protein FT EC 2.5.1.58	At3g59380	FTA (FT and GGT I common subunit)-cytosol (PLP)	No data		Very low amount of seeds	Multiple (up to 8 in a whorl) petals in a flower; homeotic transformations of flowers	Running *et al*. (2004)
	At5g40280	FTB-cytosol (ERA1; WIGGUM)	In microsporogenesis the meiosis is not synchronous and may produce aberrant tetrads or microspores, germination rate reduced	Half of the ovules do not develop into seeds	Fewer seeds than WT, development of the embryo is retarded, but not halted	Increase in floral organ number, particularly in the sepals and petals, homeotic transformations of flowers	Running *et al*. (1998); Yalovsky *et al*. (2000); Verges *et al*. (2021); Bonetta *et al*. (2000)
Protein GGT I EC 2.5.1.59	At2g39550	GGB-cytosol	Normal				Johnson *et al*. (2005); Verges *et al*. (2021)
Protein GGT II EC 2.5.1.60	At4g24490	RGTA1-cytosol	No data				Hala *et al*. (2010); Shi *et al*. (2016)
	At5g41820	RGTA2	Possibly pseudogene				
	At5g12210	RGTB1-cytosol putatively common subunit of protein GGT II and GGT III	Single mutant have fertile pollen, but lower transmission through male gametophyte; 0% genetic transmission through *rgtb1 rgtb2* pollen; single mutant pollen germinates with lower efficiency, pollen tubes often swollen, lower pollen tube guidance; *rgtb1 rgtb2* pollen have hollow phenotype; strong maternal sporophyte effect on pollen development in *rgtb1*	In heterozygote 50% lower number of ovules per silique; in homozygote 10× more ovules halted at FM to FG2 stage than in WT; strong maternal sporophyte effect on FG development	In heterozygote 10% lower seed set, in homozygote seeds develop very rarely; frequent autonomous endosperm formation in homozygotes; in homozygotes4×more deformed embryos, wider, larger and with less evident cotyledons; strong maternal sporophyte effect on seed and embryo development	Very small and retarded anthers and petals in comparison to the carpel, often homeotic transformation of terminal flowers	Gutkowska *et al*. (2015); Rojek *et al.* (2021a); Rojek *et al*. (2021b)
	At3g12070	RGTB2- cytosol putatively common subunit of protein GGT II and GGT III		Normal genetic transmission	Normal seed set, no anomalies in seed development	Normal	Gutkowska *et al*. (2015)
	At3g06540	REP- cytosol	0% genetic transmission, pollen grains develop until maturity but do not germinate	Normal genetic transmission	No homozygous progeny	n.a.	Gutkowska *et al*. (2021)
Protein GGTIII (putative)	At1g10095	PTAR homologue	n.a.				Homology to protein described in Shirakawa *et al*. (2020)
RCE1 EC 3.4.24.84	At2g36305	RCE (FACE2) endoplasmic reticulum	No data				Cadinanos *et al*. (2003); Bracha-Drori *et al*. (2008)
ICMT EC 2.1.1.100	At5g23320	ICMTA (STE14, PCM) endoplasmic reticulum	In *RNAi* lines progeny marker segregates in Mendelian fashion, meaning that gametes and seeds develop normally; increased seed dormancy			*RNAi* lines flowers developed with minimal or no elongation of the internodes, flowers developed within flowers, shoot apical meristem abnormality	Bracha-Drori *et al*. (2008); Chary *et al*. (2002); Huizinga *et al*. (2008)
	At5g08335	ICMTB (STE14, PCM) endoplasmic reticulum					
PCME EC 3.1.1.n2	At5g15860	PCME, endoplasmic reticulum	No data			Normal	Deem *et al*. (2006); Huizinga *et al*. (2008)
PCML1	At1g26120						Lan *et al*. (2010)
PCML2	At3g02410						
FCLY EC 1.8.3.6	At5g63910	FCLY, membrane fraction	No data			Normal	Crowell *et al*. (2007); Huizinga *et al*. (2010)
FLDH EC 1.1.1.216	At4g33360	FLDH, membrane fraction	No data			Normal	Bhandari *et al*. (2010)
FOLK EC 2.7.1.216	At5g58560	FOLK membrane fraction, chloroplast	Increased inhibition of seed germination by ABA			10% of flowers show homeotic transformation of organs, usually multiplication of carpel structures	Fitzpatrick *et al*. (2011a), Romer *et al*. (2024)

Only genes expressed in generative organs were included. Only enzymes with non-plastid localization were included.

#### Farnesyl diphosphate deficiency affects male gametophyte and embryo

The observed phenotypes of knock-out or knock-down of particular genes encoding for isoprenoid biosynthesis enzymes are schematically depicted in Fig. 2 and Table 1. The double mutant in both FPS-encoding genes, *fps1/fps1 fps2/fps2*, is embryo-lethal, showing a block in development at the octant/early globular stage (Closa *et al*., 2010; see Fig. 2). Detailed genetic analysis shows that a mutant *FPS1/fps1 fps2/fps2* is lethal as well (Closa *et al*., 2010). This observation may be explained by a pattern of *FPS* gene expression during embryo and seed development. While the *FPS1* gene is expressed strongly but only in the chalazal endosperm of the seed, *FPS2* is expressed in the chalazal endosperm up to the globular stage of the embryo, and inside the embryo from the early heart stage onwards (Keim *et al*., 2012). Presumably, maternal sporophytic tissues and endosperm feed the FPP or products thereof, into the developing young embryo, but FPS2 provides FPP for synthesis inside the embryo after the globular stage.

FPP molecules are precursors of sterols. The first committed reaction leading to sterols is the head to head condensation of two FPP moieties giving a branched hydrocarbon chain of squalene. This reaction is catalyzed by squalene synthase (SQS). In Arabidopsis, the only highly expressed isoform of this gene is *SQS1*. Its expression is also pronounced in all seed tissues throughout development, in the sporophytic seed coat, endosperm, and the embryo itself (https://bar.utoronto.ca/). The pattern of seed *SQS*1 expression stays in contrast to the mostly sporophytic seed coat *HMGR* and *FPS* expression described above. Hence, we hypothesize that the metabolite transported from the sporophytic integuments/seed chalaza towards the embryo up to the globular stage of development, and later from the endosperm by the apoplastic route to heart-shaped embryo, might be FPP (or farnesol). Then inside the embryo, FPP will be metabolized to squalene by SQS, and further channeled to sterol synthesis. The relatively good solubility of FPP in water corroborates this notion, but the molecular mechanisms of FPP (or farnesol, after dephosphorylation) transport from cell to cell are elusive.

However, most of the sterol biosynthesis mutants are halted later in the development, at the transition from the late globular to heart stage, so the possibility exists that the metabolite limiting the *fps1/fps1 fps2/fps2* embryo is not a sterol, but might be solanesol—a side chain in mitochondrial ubiquinone. In line with this observation is the fact that the ubiquinone biosynthesis *ppt1 (coq2)* mutant halts embryo development at the same stage (Okada *et al*., 2004; Closa *et al*., 2010).

Interestingly, total lack of FPS activity is also the cause for the highly reduced male transmission rate reaching 2.8% instead of 25% in the case of *fps1 fps2* pollen derived from *FPS1/fps1 FPS2/fps2* plants (Closa *et al*., 2010; see Fig. 2, Table 1). All pollen grains develop until maturity and germinate, in contrast to the *hmgr1 hmgr2* pollen phenotype, where pollen dies before maturation (Suzuki *et al*., 2009). Pollen grains carrying the double *fps1 fps2* mutation form slightly shorter tubes in germination tests *in vitro* and *in vivo* (Closa *et al*., 2010). This kind of pollen phenotype suggests that the metabolite missing in *fps1 fps2* pollen may be dolichol and not sterol. Similar phenotypes were described for dolichol biosynthesis mutant in the polyprenol reductase *pprd2*, where pollen grains were viable, but unable to fertilize ovules (Jozwiak *et al*., 2015). Furthermore, another mutant downstream of *pprd2*, namely *env,* mutated in a gene encoding dolichol kinase (Lindner *et al*., 2015), showed impaired genetic transmission through both male and female gametophytes. In this case, the disturbed pollen tube guidance to micropyle was responsible. Dolichols are crucial factors in protein glycosylation and GPI anchor synthesis. Both of these post-translational modifications are ubiquitous in proteins engaged in gamete recognition in plants, as well as other eukaryotes (recently reviewed in Gutkowska *et al.*, 2025a). In inter-species crosses of *Solanaceae* plants, the locus encoding farnesyl diphosphate synthase is one of the determinants of S-RNase-independent unilateral incompatibility, interestingly, from the pollen site (Qin *et al*., 2025, 2018), and we suspect that the downstream metabolite missing may be a dolichol-dependent glycosylated/GPI anchored protein involved in pollen-pistil recognition.

Genetic analysis of the *fps1 fps2* mutant reveals no defect in transmission through the female gametophyte (Closa *et al*., 2010). Strong effect of *fps1 fps2* mutation in pollen development correlates with high expression of both *FPS* genes, in particular *FPS2*, in Arabidopsis anthers and pollen grains, starting from early stages of development (Cunillera *et al*., 2000), Both *FPS* genes were also highly expressed in the stigmatic papillae and the upper part of the transmission tract, but surprisingly, low expression was observed in the ovule valves (Cunillera *et al*., 2000), as in the case of *HMGR* expression. Furthermore, wide transcriptomic screens (https://bar.utoronto.ca/) validate these experimental data obtained by FPS promotor: GUS fusion microscopic analysis. To summarize, the metabolic demand for particular classes of isoprenoids in different cells, tissues, or organs of a plant significantly differ, and hence different branches of the isoprenoid pathway are prioritized (e.g. possibly dolichol in the pollen tube and solanesol in the developing embryo).

#### Farnesol diphosphate and chloroplast development: an unexpected link

Bypassing the developmental block in *fps1/fps1 fps2/fps2* mutant, the *amiRNA* approach has been used to silence both isoforms, starting before or just after the seed germination stage (Manzano *et al*., 2016). The earlier induction of *amiRNA FPS* expression causes severe defects in chloroplast development and functioning, and leads to seedling death, while the post-germination induction of *amiRNA* expression induces chlorosis, but the seedlings survive (Manzano *et al*., 2016). Upon *FPS1* and *FPS2* silencing (*amiRNAs* used do not distinguish the isoform), chloroplast structure is severely altered, in parallel with a strong reduction in the levels of MEP pathway-derived photosynthetic pigments. Since no FPS isoform localizes to the chloroplast (Closa *et al*., 2010), and neither the FPP or its downstream metabolites, for example sterols are transported into chloroplasts (Lipko *et al*., 2023), the effect must be indirect. Presumably, chlorosis of FPP knock-downs is associated with the deregulation of genes involved in stress responses belonging to jasmonate (JA) and iron homeostasis pathways (Manzano *et al*., 2016).

Overexpression of FPP synthases in Arabidopsis (Masferrer *et al*., 2002; Manzano *et al*., 2004; Manzano *et al*., 2006) gives the same chlorotic phenotype as *FPS* silencing, which is a surprising observation. In the case of *FPS1S* overexpression, the phenotype is probably attributed to a decline in cytokinin level due to reverting the common precursor IPP from cytokinin to FPP synthesis (Fig. 1). It has been proposed that a decrease in cytokinin level causes mitochondrial dysfunction and renders plants more sensitive to oxidative damage in light-exposed chloroplasts (Manzano *et al*., 2006). This observation goes well with the fact that silencing of some genes from downstream sterol biosynthesis pathways, like cycloartenol synthase (*CAS1*), squalene epoxygenase (*SQE1*), or obtusifoliol 14-alpha-demethylase (*CYP51A2*) give similar chlorotic phenotypes (Andrade *et al*., 2017). In all these mutants the downstream pathways for farnesol utilization are inhibited, and hence the free FPP (or farnesol) may accumulate. Strikingly, other mutants more downstream in the sterol biosynthesis pathway do not show obvious chloroplast-related phenotypes. This phenotype may be also connected to general farnesol cytotoxicity, as discussed in later paragraphs.

### Geranylgeraniol function and biosynthesis in plants

#### Geranylgeranyl diphosphate– dual localization and dual metabolic role

In numerous eukaryotes geranylgeranyl diphosphate is synthesized, similar to FPP, in the cytoplasm. In plants, the situation is not that obvious. The main GGPP pool is produced in the chloroplast from the MEP pathway-derived precursors (Wang *et al*., 2023; Fig. 1). In chloroplasts, GGPP is a precursor for photosynthetic pigments: chlorophyll side chains, carotenoids, and other important chloroplast membrane constituents such as plastoquinone, tocopherols or long chain isoprenoid alcohols; but this issue is beyond the scope of this review (Ruiz-Sola *et al*., 2016b; Akhtar *et al*., 2017; Lipko *et al*., 2023; see Fig. 1). Apart from primary metabolism, chloroplast-produced GGPP is a source of a plethora of secondary metabolites from the diterpene group, playing roles in plant-pathogen interactions, abiotic stress responses, pollinator and herbivore interaction, and many others, beyond the subject of this work (Zi *et al*., 2014; Wang *et al*., 2023).

Nevertheless, a pool of GGPP must also be present in the cytoplasm and mitochondria, where it serves for the synthesis of side chains of ubiquinone and protein prenylation (Fig. 1). Whether GGPP is produced *in situ* in the cytoplasm from MVA-derived precursors or it is imported from plastids (of MEP origin), remains a matter of a long debate (Ruiz-Sola *et al*., 2016a; Vranova *et al*., 2019; Chevalier *et al*., 2024). For example, the plastidial MEP pathway provides the isoprenyl moiety for protein geranylgeranylation in tobacco BY-2 cells, but this model, convenient for biotechnology, may be treated as somehow artificial (Gerber *et al*., 2009; Chevalier *et al*., 2024). Another unanswered question is in what form (as a phosphate ester or free alcohol) geranylgeraniol may be transported into or out of chloroplasts inside the cell or from cell to cell. Nevertheless, together with IPP, GG(PP) remains the main candidate for short-chain isoprenoid transport (Skorupinska-Tudek *et al*., 2008; Gerber *et al*., 2009; Huchelmann *et al*., 2016; Jozwiak *et al*., 2017; Vranova *et al*., 2019; Lipko *et al*., 2023; Romer *et al*., 2024, see Fig. 1).

#### Geranylgeranyl diphosphate synthase family in Arabidopsis

The 20-carbon geranylgeranyl diphosphate tri-*trans*(E,E,E)*-*GGPP is synthesized from one molecule of DMAPP and three molecules of IPP in consecutive head-to-tail condensation reactions catalyzed by geranylgeranyl diphosphate synthase GGPS (Fig. 4). Similar to FPS enzyme, the GGPS is a hydrophilic protein, and both are structurally and evolutionary related (Chang *et al*., 2021). The protein fold of FPS and GGPS is strikingly similar, differing mainly in the active site depth accommodating shorter (FPP) or longer (GGPP) products (Ohnuma *et al*., 1996).

In plants, all-*trans*(E)-GGPP synthases encoding genes usually constitute families of several members, and most of their protein products are localized to chloroplast stroma (Okada *et al*., 2000; Beck *et al*., 2013; Nagel *et al*., 2015). These gene families are larger in the species producing specialized diterpenes (e.g. *Pinaceae*, *Lamiaceae*) and smaller in plants with less diverse diterpene metabolism (Coman *et al*., 2014). In Arabidopsis, five *GGPS* genes encode enzymes specifically producing only GGPP, and no other related compounds such as geranyl diphosphate (GPP, 10 carbon atoms) or geranylfarnesyl diphosphate (GFPP, 25 carbon atoms) (Beck *et al*., 2013; Nagel *et al*., 2015; You *et al*., 2020). These are *GGPS3* and *GGPS4* whose protein products localize to cytoplasm/ER; *GGPS1* is putatively the mitochondrial isoform, and proteins encoded by *GGPS2* and *GGPS11* localize to the chloroplast (Okada *et al*., 2000), the latter being the predominant chloroplast isoform (Ruiz-Sola *et al*., 2016b; Table 1). Recently, it has been shown that the short isoform of GGPS11 (called GGPS11S) is the main cytoplasmic isoform as well (Ruiz-Sola *et al*., 2016a). The role of GGPS3 and GGPS4 in the cytosol, or GGPS1 in the mitochondria remains unclear, but any of them might be generating GGPP for protein geranylgeranylation or ubiquinone side chain synthesis (Beck *et al*., 2013; Ruiz-Sola *et al*., 2016b). A summary of the studies on GGPP synthases in Arabidopsis has been recently presented (Kopcsayova and Vranova, 2019).

#### *Mutants of all-*trans*(E)-geranylgeranyl diphosphate synthases: geranylgeranyl diphosphate 11 (GGPS11) as the major player in chloroplast and cytosolic geranylgeranyl diphosphate metabolism*

Initially characterized mutants in the *GGPS11* gene in Arabidopsis showed only mild variegation of the leaves (Ruppel *et al*., 2013) or albino-seedling lethal phenotype (Ruiz-Sola *et al*., 2016b), which is consistent with the role of *GGPS11* in chloroplast photosynthetic pigment synthesis. *GGPS11* can also give rise to a shorter translational isoform, devoid of the chloroplast localization signal. This form called *GGPS11S* has recently been established to be the main cytosolic source of GGPS activity (Ruiz-Sola *et al*., 2016a). The GGPS11S-deficient embryo is halted in development at the early heart stage (Fig. 2) (Ruppel *et al*., 2013; Ruiz-Sola *et al*., 2016a; Table 1). Supplementation with GGPS11S isoform reverts only the embryo lethality, but the seedling is still unable to establish photosynthesis (Vranova *et al*., 2019). Of note is that *GGPS11* is expressed in the maternal tissues surrounding the embryo at early stages of development, but very low expression is detected inside the embryo until it reaches the heart stage (Beck *et al*., 2013). This observation further confirms that the sporophytic tissues of the ovule feed the developing embryo with GGPP, similar to what is observed with FPP. Another explanation of the *ggps11-4* mutant embryo lethality is, that the embryo cannot produce geranylgeraniol used for chlorophyll synthesis starting from the early heart stage and establish its own photosynthetic activity (Tejos *et al*., 2010; Sela *et al*., 2020). Pollen of the *ggps11* mutant, in opposition to the *fps1 fps2* mutant, must be fertile, as it is possible to obtain a Mendelian segregation in *GGPS11/ggps11* plant progeny (Ruiz-Sola *et al.,* 2016b). It means that GGPP synthase other than GGPS11 provides prenyl residues for protein geranylgeranylation in growing pollen tubes.

#### Mutants of other cytoplasmic all-trans(E)-geranylgeranyl diphosphate synthases

Single mutant lines for any of the other *GGPS* genes, expressing chloroplast, cytosolic, or mitochondrial GGPPS, are viable (Ruiz-Sola *et al*., 2016b), and their activity is redundant. Only mitochondrial GGPS1 and cytosolic GGPS4 are expressed in the seeds or flower stamens, respectively (Beck *et al*., 2013; Table 1). The *ggps1* mutant has no deviation from the Mendelian inheritance pattern, contains a normal level of ubiquinone, and presents no developmental defect (Ruiz-Sola *et al*., 2016b) which raises the possibility that it is not responsible for the synthesis of the isoprenoid anchor for ubiquinone, or that in case of GGPP shortage, the ubiquinone may be modified by a prenyl chain derived from FPP. More detailed characteristics (and genetic segregation analysis) of the *ggps4* mutant are missing, but it might be presumed, that its high expression in anthers (Beck *et al*., 2013; https://bar.utoronto.ca/) plays a role in pollen development constitutively or in *GGPS11S* knock-outs. A high need for cytoplasmic geranylgeranyl diphosphate for Rab protein prenylation (see later paragraphs) cannot be ensured upon a total lack of GGPS activity in germinating pollen and fast-growing pollen tubes. The assumption of GGPS4 being a main pollen isoform awaits experimental proof.

## Protein prenylation

### Function of protein prenylation—more than a sticky glue

Protein prenylation is a process in which the geranylgeranyl or farnesyl moiety of GGPP or FPP is covalently attached to a cysteine residue near the C-terminus of the peptide chain (Wang and Casey, 2016; see Fig. 4). This leads to the formation of a chemically stable thioether bond that practically precludes *in vivo* deprenylation of proteins, as the prenyl group may be detached only after protein degradation (Huizinga *et al*., 2010; Barone *et al*., 2024). The process of protein prenylation occurs in all eukaryotes and its main purpose is to attach a hydrophobic farnesyl or geranylgeranyl lipid anchor to an otherwise soluble protein to ensure its peripheral membrane attachment (Fig. 3). The *in vitro* studies show that peptides farnesylated on C-terminal cysteine residues exhibit affinity to lipid vesicles of 50–400 μM (lower than myristoylated peptides) and geranylgeranylated of 2–8 μM (approximately five times lower affinity than palmitoylated peptides) (Silvius and l'Heureux, 1994; Shahinian and Silvius, 1995). The half-time of membrane-membrane transfer (approximation of affinity) of peptides modified with two neighboring GG groups reaches 10 h *in vitro*, while mono-*S*-acylated and mono-prenylated lipopeptides insert into, and spontaneously transfer between lipid vesicles on a very rapid time scale of seconds (Shahinian and Silvius, 1995).

Apart from the membrane anchoring role, the prenylation may constitute a part of the protein-protein recognition motif (Zhang *et al*., 2018; Zhou and Hancock 2018; 2020; Bezeljak *et al*., 2020; Lee *et al*., 2020). This posttranslational modification recognizes high lipid membrane curvature or lipid packing order defects (Beck *et al*., 2008; Li *et al*., 2012; Kirsten *et al*., 2013; Kulakowski *et al*., 2018; Maxwell *et al*., 2018). Prenyl groups may even introduce local mis-organization to the lipid bilayers (Janosi and Gorfe, 2010; Janosi *et al*., 2012). All-*trans*(E)*-*prenyl groups show higher affinity for structurally disordered domains of the membrane *in vitro*, and consequently, prenylated peptides and proteins are considered to be excluded from sterol-enriched micro-domains (Levental *et al*., 2010; Janosi *et al*., 2012; Kulakowski *et al*., 2018; see Fig. 3). It has been suggested that proteins dually modified with prenyl and acyl groups reside at the edge of sterol-enriched membrane microdomains (Levental *et al*., 2010; Fig. 3). Prenyl groups which are attached to proteins recognize phosphatidylserine preferentially with one acyl chain saturated and one unsaturated (Zhou *et al*., 2021).

### Protein prenylation or geranylgeraniol as a negative feedback input for isoprenoid biosynthesis

In non-plant Eukaryotes with decreased MVA pathway efficiency, the symptoms of a decrease in protein prenylation seem to occur earlier than deficiency in any other isoprenoid, e.g. sterols or ubiquinone (Callegari *et al*., 2010; Liscum, 2011; Fracassi *et al*., 2018). It seems therefore that the prenylation of proteins is prioritized over sterol biosynthesis, at least in mammalian tissues analyzed so far (e.g. Houten *et al*., 2003) and in unicellular yeasts as well (Callegari *et al*., 2010). Whether protein prenylation in some plant tissues is preferential to sterol biosynthesis needs to be elucidated in further studies.

The tuning of whole MVA pathway intermediates flow by parallel feedback inputs from GGPP (or geranylgeranylated proteins) and sterols on HMGR activity has emerged as an important subject of study in yeast and animals (Garza *et al*., 2009; Leichner *et al*., 2011; Theesfeld and Hampton, 2013; Schumacher *et al*., 2015; Wangeline and Hampton, 2018, 2021; Elsabrouty *et al*., 2021; reviewed in Erffelinck and Goossens, 2018). Geranylgeraniol-induced HMGR proteolytic degradation in mammals and yeast uses different molecular mechanisms to achieve common results—down-regulation of the production of isoprenoid precursors for protein prenylation and sterol biosynthesis. Whether geranylgeraniol or its derivative exerts such an effect in plants seems plausible (Gutkowska *et al.*, 2025b). Taking into account the vigorous geranylgeraniol metabolism inside chloroplasts Ruiz-Sola *et al*. (2016b) and the possible geranylgeraniol transport from chloroplasts to the cytoplasm (Huchelmann *et al*., 2016), the involvement of a geranylgeranylated protein seems more plausible as a signal for HMGR regulation.

### Protein prenyltransferases—a patchwork family of enzymes

**Fig. 5.**
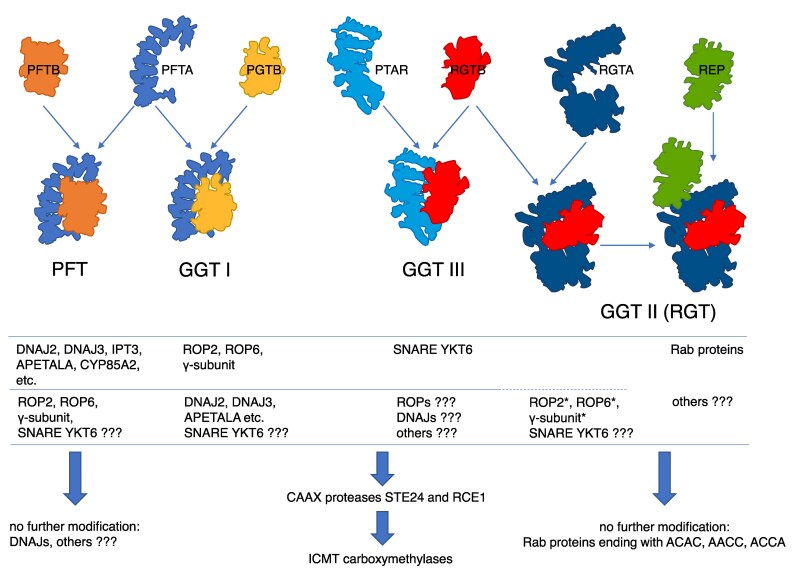
Schematic representation of protein prenyltransferases subunit composition and protein substrate specificity. Subunit organization and three-dimensional structure of protein prenyltransferases is based on human protein structures presented in Marchwicka *et al*. (2022). Homologous subunits are represented in different colors. Note general similarity of folds for *α* and *β* subunits of protein prenyltransferases. Experimentally confirmed native protein substrates shown in upper row of the table, non-native substrates confirmed experimentally *in vivo* shown in the middle row of the table, asterisks denote experimentally confirmed *in vitro* substrates. In the lower row of the table putative substrates are shown with question marks. Abbreviations: GGT1, protein geranylgeranyl transferase I; GGT III, putative protein geranylgeranyl transferase III; GGT II (RGT), protein geranylgeranyl transferase II (Rab geranylgeranyl transferase); PFT, protein farnesyltransferase; PFTA, alpha subunit of PFT and GGT1; PFTB, beta subunit of PFT; PGTB, beta subunit of GGT1; PTAR, putative protein geranylgeranyl transferase III alpha subunit; REP, Rab escort protein; RGTA, geranylgeranyl transferase II alpha subunit; RGTB, geranylgeranyl transferase II and III beta subunit.

In *Arabidopsis thaliana* so far three protein prenyltransferases have been described. These are protein farnesyltransferase (FT) (Yalovsky *et al*., 2000; Ziegelhoffer *et al*., 2000), protein geranylgeranyltransferase I (GGT I) (Johnson *et al*., 2005), and Rab protein geranylgeranyl transferase (RGT), also known as protein geranylgeranyl transferase II (GGT II) (Hala *et al*., 2010). In other eukaryotes, the fourth protein prenyltransferase has been recently reported (GGT III) (Shirakawa *et al*., 2020; Sakata *et al*., 2021; Tateishi *et al*., 2025), and its existence in plants is highly likely (Tateishi *et al*., 2025). All these enzymes consist of two subunits: α- catalytic subunit (FTA) and β-lipid substrate presenting subunit (FTB and GGB) (Fig. 5). The FT and GGT I share the same α- subunit, but have distinct, but highly similar β- subunits (reviewed recently by Marchwicka *et al*., 2022). RGT has its own α- subunit (RGTA) that has generally similar backbone fold as in the other protein prenyltransferases; the β subunit (RGTB) is probably shared with the GGT III enzyme (Shirakawa *et al*., 2020; Marchwicka *et al*., 2022; Tateishi *et al*., 2025).

FT and GGT I are evolutionarily and structurally related, and recognize their protein targets based only on short, 4- or 5-amino acid long motifs—CAAX, or recently discovered CAAAX (Schey *et al*., 2024)—at the C-terminus of the proteins; yeast enzymes are also able to recognize shorter or non-C-terminal motifs *in vivo* and *in vitro* (Berger *et al*., 2018; Ashok *et al*., 2020). The typical motif recognized by GGT I is CAAL and by FT the most typical motifs are CAAS, CAAA, CAAM, and CAAC. FT, and GGT I enzymes are redundant to a wide extent. Plant and other eukaryotic FT enzymes can catalyze a transfer of farnesyl group, instead of geranylgeranyl, onto a cysteine of CAAL geranylgeranylation motif equally well as typical CAAM or CAAS farnesylation motifs *in vitro* (Andrews *et al*., 2010; Krzysiak *et al*., 2010) and *in vivo* (Zeng *et al*., 2007; Sorek *et al*., 2011; Stein *et al*., 2015; Berger *et al*., 2018). Protein FT can prenylate most GGT I substrates *in vitro* and in yeast cells, but usually cannot use GGPP as a prenyl group donor (Reid *et al*., 2004; Ashok *et al*., 2020). In contrast, the GGT I enzyme can modify typical farnesylation targets *in vitro* (Trueblood *et al*., 1993; Hartman *et al*., 2005; Hicks *et al*., 2005; Andrews *et al*., 2010). Altogether more than 700 proteins meet the strict criteria of prenylation in Arabidopsis, as judged by a bioinformatic search (Galichet and Gruissem, 2003), and many more fit to the non-canonical ones. The list of experimentally confirmed protein farnesylation and geranylgeranylation targets in plants is nevertheless surprisingly limited (Hala and Zarsky, 2019). The biochemical perspective on CAAX protein prenyltransferases in plants has been recently presented (Chevalier *et al*., 2025).

The proteins targets of FT in plants belong to diverse groups and diverse sub-cellular locations: nuclear calmodulin (Rodriguez-Concepcion *et al*., 1999), plasma membrane heavy metal binding proteins (Gao *et al*., 2009), rough endoplasmic reticulum Hsp40 chaperone homologs (J2 and J3; Barghetti *et al*., 2017), nuclear floral transcription factor APETALA1 (Yalovsky *et al*., 2000), endoplasmic reticulum cytochrome P450 CYP85A2 involved in brassinosteroid biosynthesis (Northey *et al*., 2016), nuclear/cytoplasmic IPT3 enzyme involved in cytokinin biosynthesis (Galichet *et al*., 2008) and others—for the full list of experimentally confirmed farnesylated proteins see recent reviews (Hala and Zarsky, 2019; Tian and Wang, 2025). Of note is that in many cases, the farnesylation signal is not demanded for membrane insertion, but is crucial for protein localization and function (Rodriguez-Concepcion *et al*., 1999; Yalovsky *et al*., 2000). GGT I targets are less varied and mainly belong to small GTPases like Rho of plants (ROPs) (Chai *et al*., 2016), and γ-subunit of trimeric G-proteins (Zeng *et al*., 2007). In the case of these proteins, the second lipidation (usually palmitoylation) is often demanded for stable lipid anchorage to the membrane (Zeng *et al*., 2007; Sorek *et al*., 2011; Fig. 3).

RGT differs from GGT I in that it needs an obligatory accessory subunit for prenylation, Rab Escort Protein (REP), which presents the substrates, Rab proteins, for catalysis by RGTA/RGTB dimer (reviewed in Gutkowska and Swiezewska, 2012). The function of REP in Rab prenylation is to recognize the Rab protein and escort it to the RGT enzymatic complex. *In vitro*, RGT enzyme, without involvement of REP, can prenylate some GGT I targets (Shi *et al*., 2016). Some Rab proteins, possessing CAAX amino acid motifs, may be recognized *in vitro* and be prenylated by the GGT I enzyme, while others cannot. A total lack of RGT or REP activity is lethal for a plant (Gutkowska *et al*., 2015, 2021; Fig. 2, Table 1).

#### Mutants in the β subunit of protein farnesyl transferase

The most striking phenotypes of the *era1* mutant in the β-subunit of the protein farnesyl transferase (FTB) are connected with the organization of the shoot and floral meristem. Plants are viable, but the sporophytic phenotype is strong, in particular in the short day conditions (Yalovsky *et al*., 2000). The *era1* (*wig1*) alleles of the FTB, both knock-out and point mutations, are fertile, but have enlarged meristems and floral organs, and homeotic transformations of flowers that often fail to produce any reproductive organs (Running *et al*., 1998; Yalovsky *et al*., 2000; Verges *et al*., 2021; Fig. 2, Table 1). *era1* mutants produce fewer but larger seeds than wild type (Verges *et al*., 2021). *era1* microsporogenesis is strongly perturbed, the meiosis is not synchronous, and aberrant tetrads or degenerated microspores are produced, reducing the quality of the pollen production (Bonetta *et al*., 2000; Verges *et al*., 2021). About half of the ovules do not develop into seeds in *era*1 (Verges *et al*., 2021). *era1* developmental phenotypes are summarized in Fig. 2 and Table 1. The phenotypes of *era1* Arabidopsis plants have been attributed to reduced prenylation of the transcription factor APETALA1 or Hsp40 chaperone J3 (Barghetti *et al*., 2017). Interestingly, in the latter case, the farnesyl cannot be substituted by geranylgeranyl by the action of GGT, probably because GG anchor is not a correct recognition signal for a protein effector of J proteins. Farnesylation of DNAJ-like Hsp40 proteins J2 and J3 in Arabidopsis facilitates Argonaute complex binding to endoplasmic reticulum membranes, and the formation of small RNAs (Sjogren *et al*., 2018). Hsp40 chaperone is also involved in the maturation of a plethora of different proteins (Barghetti *et al*., 2017). Therefore, the lack of farnesylation of J2 and J3 may infer complex pleiotropic phenotypes in plants, depending on DNA J chaperoned substrates or the release of small RNAs.

The second group of observable phenotypes connects the *era1* response to the abscisic acid (ABA) treatment. The Arabidopsis *era1* mutant is hypersensitive to ABA and shows a delay in germination as well as decreased stomatal opening, hence its name ‘enhanced response to ABA’ (Cutler *et al*., 1996). The trait of reduced stomatal gas exchange has been used to construct drought-resistant *Canola* plants (Wang *et al*., 2005, 2009). Rice *era1* mutant lines, harboring CRISPR/Cas9-induced frameshift mutations, exhibit similar leaf growth as control plants but increased primary root growth. The rice *era1* mutant lines also display increased sensitivity to ABA and an enhanced response to drought stress through stomatal regulation (Ogata *et al*., 2020), similar to soybean (Ogata *et al*., 2017). In multicellular moss *Physcomitrium patens*, knock-out of *FTB* has a relatively mild phenotype. The cells differentiate slower, and grow more round than in wild type, but generally, the plant keeps their normal body plan; however, the impaired differentiation of the cells makes the plants infertile (Thole *et al*., 2014). In *P. patens* it seems that the GGT enzyme substitutes quite efficiently for FT in the sporophyte, in comparison to the FT/GGT pair in Arabidopsis (Johnson *et al*., 2005).

#### Mutants in the β subunit of protein geranylgeranyl transferase I

Mutants in the *β* subunit in geranylgeranyl protein transferase I (GGB) in Arabidopsis show ABA and auxin-related phenotypes in the sporophyte, such as decreased stomatal opening, adventitious root formation, and delayed seed germination (Johnson *et al*., 2005), but not in the gametophyte or seed formation (Verges *et al*., 2021; Table 1). The Rho family (ROP2 and ROP6) proteins or γ-subunit of heterotrimeric G-proteins are candidates for the hypo-geranylgeranylated proteins in the *ggb* mutant (Lemichez *et al*., 2001; Subramaniam *et al*., 2016). Type ROP I proteins (ROP2 and ROP6 included) are preferentially geranylgeranylated *in vivo* and *in vitro* in wild type plants, but in the *ggb* knock-out mutant are farnesylated. Because they carry a secondary palmitoylation signal, the presence of a farnesyl group instead of geranylgeranyl has a negligible effect on protein functions (Sorek *et al*., 2011). The overlapping specificity of GGB and FTB probably explains the relatively weak phenotype of *ggb* in Arabidopsis in comparison to strong polarity and differentiation defects of *GGB* knock-out in the moss, *Physcomitrium patens.* Due to differentiation defects plants of the moss are infertile, the ROP protein affected by lack of GGB is farnesylated by FT, but this does not increase its hydrophobicity enough to interact efficiently with the membrane (Thole *et al*., 2014; Bao *et al*., 2022).

#### Mutants in common CAAX prenyltransferases subunit

The knock-out allele of the common α subunit shared by FT and GGT (FTA) was given the name *pluripetala* (*plp*) due to the striking phenotype of multiple (up to eight in a whorl) petals in a flower (Yalovsky *et al*., 2000; Table 1). The *plp* plants are fertile through both male and female lines, but produce a lower amount of seeds than wild type or even *era1* mutants (Running *et al*., 2004). Flowers of *plp* have an increased organ number with more sepals, petals, and sometimes carpels, and the stamens and carpels are larger in size (Running *et al*., 2004). The delocalization of ROP proteins has been observed in the *plp* Arabidopsis mutant, leading to defective growth of root hairs (Chai *et al*., 2016).

The Arabidopsis double mutant with both β-subunits of protein prenyltransferases FT and GGT I knocked-out (*era1 ggb*) is indistinguishable from the *plp* mutant (Running *et al*., 2004). It is striking that Arabidopsis plants devoid of both FT and GGT I activity survive at all. Interestingly, the RGT enzyme (without REP) was shown to perform prenylation of ROP and γ-subunit of heterotrimeric G-proteins *in vitro*, natively being the GGT I substrates (Shi *et al*., 2016). Whether such modification is possible *in vivo*, or if the putative GGT III prenyltransferase is active towards GGT I targets remains an open question. In mammals, GGT III transfers a geranylgeranyl group to monofarnesylated Soluble NSF Attachment Protein REceptor (SNARE) Ykt6, generating doubly prenylated Ykt6 (Shirakawa *et al*., 2020; Sakata *et al*., 2021) or to ubiquitin ligase (Kuchay *et al*., 2019). The same enzymatic activity has been reported in yeast (Tateishi *et al*, 2025). These observations, together with the discovery of new prenylation motifs for CAAX protein prenyltransferases (Berger *et al*., 2018; Ashok *et al*., 2020; Schey *et al*., 2024) raise a possibility that the exchange of substrates between protein prenyltransferases is higher than was suspected. Proteomic studies with new bio-orthogonal prenyl analogs and the use of protein prenyltransferase inhibitors should help in the elucidation of the specificity of the newly discovered GGT III (Storck *et al*., 2019; Justyna *et al*., 2023; Auger *et al*., 2025). The activity of GGT III is not confirmed in plants, but the survival of the Arabidopsis *plp* mutant in comparison to lethal defects in *plp* mutant in *P. patens*, where cells are unable to form colonies or differentiate, may arise from different cross-reactivity between this enzyme and GGT I (Thole *et al*., 2014). Data presented in Tateishi *et al.* (2025) allow to pre-identify *At1g10095* as the prenyl protein transferase III α-subunit (PTAR)-encoding gene in Arabidopsis.

#### Mutants in protein geranylgeranyl transferase II complex affect male and female gametophyte development

Rab geranylgeranyl transferase (RGT) stands out from the other protein prenyltransferases in its necessity of an accessory Rab Escort Protein (REP) in the process of geranylgeranylation (Fig. 5). The RGT complex consists of tightly bound α- and β-subunits, with the catalytic site located in the α- and prenyl phosphate lipid binding in the β-subunit (reviewed in Gutkowska and Swiezewska, 2012; Marchwicka *et al*., 2022). Rab protein substrate is recognized by Rab Escort Protein by the interaction of protein surfaces distant from the prenylatable cysteines and presented for prenylation. In plants, the amino acids engaged in Rab-REP recognition are invariant on REP, but the contribution of different motifs in different Rab families is observed (Gutkowska *et al*., 2021). REP holds Rab proteins during two consecutive geranylgeranyl additions, and only then dissociates from the RGT complex (Thoma *et al*., 2001). The REP surface provides a hydrophobic pocket for prenylated cysteines and delivers Rab to a destination membrane. The prenylatable cysteines on Rab proteins are present near the C-terminus of the substrate in adjacent positions: -CCXX, -XCXC, -XXCC, -XCCX in plant Rabs also in -CCXXX. Dual prenylation of cysteines is necessary for correct Rab localization and function, as shown in yeast (Calero *et al*., 2003; Gomes *et al*., 2003). The functions of Rab proteins in plants include polarity establishment, cell wall biosynthesis and modification, response to pathogens, and have been recently reviewed (Martiniere and Moreau, 2020; Nielsen, 2020; Fu *et al*., 2025).

Arabidopsis RGT α subunit (RGTA) is encoded by two genes, but one of them gives a product with very low, if any, enzymatic activity, as was shown in a broad *in vitro* study (Shi *et al*., 2016); therefore only *RGTA1* may be recognized as a functional gene. RGT β subunit (RGTB) is encoded by two highly homologous, partially redundant genes, of which *RGTB1* is ubiquitously expressed both in sporophyte and gametophyte, and *RGTB2* expression is weaker and restricted to some tissues only (Hala *et al*., 2010; Gutkowska *et al*., 2015). REP protein is encoded by a single gene (Hala *et al*., 2005).

Several mutants in the RGT complex in Arabidopsis have been described (Hala *et al*., 2010; Gutkowska *et al*., 2015; Gutkowska *et al*., 2021; Rojek *et al*., 2021a; Rojek *et al*., 2021b). A mutation in *RGTB1* leads to pleiotropic phenotypic deviations such as reduced plant growth, increased branching, delayed senescence, loss of shoot gravitropic response, de-etiolation of the seedling, short and branched root-hairs and compromised exo- and endocytosis (Hala *et al*., 2010; Gutkowska *et al*., 2015). Concerning pollen development RGT activity seems indispensable, since the T-DNA mutants in both β subunit encoding genes *rgtb1* and *rgtb2* have reduced male fertility (Fig. 2, Table 1) due to pollen tube malformations (Gutkowska *et al*., 2015). *rgtb1* mutant is additionally retarded in the development of anthers (Hala *et al*., 2010; Gutkowska *et al*., 2015) and has reduced pollen tube guidance (Rojek *et al*., 2021b). Double *rgtb1 rgtb2* mutant pollen is sterile due to male gametophyte defects (Gutkowska *et al*., 2015; Fig. 2, Table 1). Homozygous *rgtb1* mutants are also show low female fertility—25% of ovules become arrested at the FM/FG2 stage of development due to abnormal auxin accumulation inside the embryo sac, a maternal sporophytic defect of gametophyte development (Rojek *et al*., 2021a). Homozygous *rgtb1* plants fertilized with wild type pollen form fewer seeds than controls, and more often produce autonomous endosperm (Rojek *et al*., 2021b). Even if the embryo is formed it is often larger and somehow misshapen due to disturbed transport of auxins inside the seed (Rojek *et al*., 2021b; Fig. 2, Table 1). In *Physcomitrium patens* two genes encoding RGTB are present, with the enzymes having redundant functions; hence the phenotype of the single mutant is not changed in comparison to wild type (Thole *et al*., 2014). Similar to Arabidopsis, the *P.patens* double mutant is lethal (Thole *et al*., 2014), suggesting that GGT I is unable to prenylate some Rab proteins essential for plant survival (Fig. 5).

No mutants in the *RGTA1* gene have been described so far in Arabidopsis and *Physcomitrium*, probably due to their gametophyte lethality; however, the knock-out of the *REP* gene in Arabidopsis is known (Gutkowska *et al*., 2021). In the case of this mutant, the *rep* knock-out allele is not transmitted by the male line, pollen grains are viable, but unable to germinate (Gutkowska *et al*., 2021; Fig. 2). In *P. patens* the situation is the same—REP activity is single-gene encoded and its knock-out is non-viable (Thole *et al*., 2014).

### Post-prenylation processing of the proteins

#### Proteolytic removal of -AAX peptide

After being modified by a farnesyl or geranylgeranyl moiety some proteins undergo post-translational processing by proteolytic removal of -AAX C-terminal sequence (reviewed in Wang and Casey, 2016; Fig. 4). This reaction in Arabidopsis is catalyzed by prenylcysteine-specific but structurally unrelated proteases—RCE1 (Ras Converting Enzyme) and FACE1/STE24 (Factor α Converting Enzyme/ STErile24), each encoded by a single gene (Bracha *et al*., 2002; Cadinanos *et al*., 2003). Many FT and GGT I products are processed in this manner, and the incidence of the modification depends on amino acid residues adjacent to prenylated cysteine both upstream and downstream, in the -AAX sequence (Berger *et al*., 2018). RCE1 and STE24 have distinct but overlapping substrate specificity (Cadinanos *et al*., 2003). In contrast to protein prenyltransferases which are soluble enzymes, prenylated peptide-specific proteases are ER membrane-embedded proteins (Bracha-Drori *et al*., 2008).

#### Methylation of the prenylated cysteine

C-terminal prenylated cysteine released as a result of the proteolytic cleavage (FT or GGT I targets, also Rab proteins with -CAAX motif; Leung *et al*., 2007) may undergo further post-translational processing, via methylation by (iso)prenylcysteine methyl transferase enzyme ICMT (reviewed in Wang and Casey, 2016; Fig. 4). In this reaction, a C-terminal methyl ester with the carboxyl group of cysteine residue is formed. This step is protein-sequence independent, but vulnerable to the length of the lipid chain, i.e*. in vitro* only farnesylated or geranylgeranylated cysteine is a substrate, and the enzyme does not use shorter or longer isoprenoid modified cysteines efficiently (Ma *et al*., 1995). The addition of methyl group on protein C-terminal ionized carboxyl group increases the hydrophobicity and changes the affinity of prenylated cysteine towards membranes by more than 10 times in *in vitro* assays (Silvius and l'Heureux, 1994; Fig. 3). What is more surprising is that the prenylcysteine methylation correlates with the plasma membrane localization of proteins in plants (Rodriguez-Concepcion *et al*., 2000; Bracha-Drori *et al*., 2008), while ICMT enzyme is ER-membrane localized (Bracha-Drori *et al*., 2008). While post-prenylation processing appears to be required for proper membrane localization of farnesylated proteins (e.g. Ras in mammals), it is not required for membrane targeting and function of geranylgeranylated Rho GTPases, at least in animal cells (Michaelson *et al*., 2005). In some cases, like in yeast, the post-prenylation processing is even detrimental for the protein function (Hildebrandt *et al*., 2016; Berger *et al*., 2018). No data are available for plants in this respect.

The prenylated cysteine methylation enzymatic step was first described in tobacco (Crowell *et al*., 1998) and later in Arabidopsis, where it is catalyzed by products of two related genes: *ICMTA* and *ICMTB* (Rodriguez-Concepcion *et al*., 2000; Chary *et al*., 2002) encoding prenyl cysteine methyl transferases. The *ICMT* genes in Arabidopsis differ in the expression sites and level, with *ICMTB* being the more abundant and ubiquitously expressed (Chary *et al*., 2002). Inhibition of ICMT activity reduces the germination rate of seeds and other ABA-related processes such as stomata closure (Chary *et al*., 2002), Table 1. The phenotype of the *ICMT RNAi* lines in Arabidopsis is similar to the farnesyltransferase β-subunit mutant *era1* (Bracha-Drori *et al.,* 2008). Flowers of the *icmt* mutant show floral homeotic transformation and bushy stature of the plant (Bracha-Driori et al., 2008). Contrary to the *era1* mutant, no flower organ multiplication is observed in the *icmt* mutant (Bracha-Drori *et al*., 2008).

#### Demethylation of the prenylated cysteine

Farnesylation or geranylgeranylation of proteins involves the formation of a very stable thioether bond between the thiol group of the cysteine and a hydroxyl group of the isoprenoid alcohol. So far no enzymes able to release this modification from intact proteins have been identified, and it seems that the farnesyl or geranylgeranyl group may be released only from free prenylated cysteine after proteolytic degradation of the peptide (Lu and Hofmann, 2006, Fig. 4). Hence the only reversible reaction of protein processing by isoprenoids is the removal of the carboxy-terminal methyl group. (Iso)Prenylcysteine methylesterase ICME activity was found in many eukaryotes, including plants (Deem *et al*., 2006).

In Arabidopsis three genes responsible for prenylcysteine demethylation have been characterized: *ICME*, *ICMEL1*, and *ICMEL2* (Huizinga *et al*., 2008; Lan *et al*., 2010). Not surprising is the fact that the phenotypes of *ICME* knock-out are opposite to *ICMT* knock-outs, and include lower sensitivity to ABA in stomatal closure and seed germination (Chary *et al*., 2002; Deem *et al*., 2006). Overexpression of *ICME* renders plants hypersensitive to ABA and overexpression of *ICMT* hyposensitive to ABA (Huizinga *et al*., 2008). This means that the geranylgeranylated protein(s) responsible for ABA responses are also further processed by proteolysis and methylation/demethylation. These proteins probably belong to the Rho family or γ-subunit of heterotrimeric G-proteins (Lemichez *et al*., 2001; Subramaniam *et al*., 2016). Contrary to the *plp* mutant in the α-subunit of farnesyl/geranylgeranyl transferase, *icmt* or *icme* mutants show no homeotic transformation of the flower organs (Table 1). It has been recently shown that the hypo prenylation of chaperone Hsp40 (DNAJ homologue) might be responsible for the inflorescence and floral meristem transformation in *plp* mutants (Barghetti *et al*., 2017). It is possible that, similar to its yeast counterpart, plant Hsp40 J3 is farnesylated, but does not undergo any further processing at the C-terminus (Hildebrandt *et al*., 2016; Berger *et al*., 2018).

### Degradation of prenylated proteins

Prenyl groups from proteins is released only after peptide degradation, while palmitoyl modification on proteins is reversible and plays a regulatory role (Hemsley, 2020). In animal cells, Rab guanine nucleotide dissociation inhibitor (GDI), a protein involved in Rab recycling, interacts with Hsp90 and this chaperone promotes recycling of Rab proteins (Chen and Balch, 2006). Hsp90 serves not only as a folding factor but also as a proteasome-directing protein (reviewed in Prodromou, 2023), and this interaction may be conserved in plants as well. Prenylated protein degradation in non-plant eukaryotes (in the proteasome) leads to the release of free prenylated cysteine. Farnesyl and geranylgeranyl groups must be then detached from cysteine residues to form prenal aldehydes, which are hydrogenated and later phosphorylated for their recycling or degradation (in the medical context reviewed in Verdaguer *et al*., 2022).

### Farnesol (or geranylgeraniol) salvage pathway

#### Deprenylation of modified cysteine

The thioether bond between the prenyl moiety and free amino acid cysteine may be broken by an enzyme prenylcysteine lyase (FCLY), present in plants and other eukaryotes (Crowell *et al*., 2007, recently reviewed in Hemmerlin, 2023; Fig. 4, Table 1). This enzyme cleaves lipids from a cysteine producing a prenyl aldehyde (i.e. farnesal and geranylgeranial). Prenyl cysteine lyase is NAD(P)H-independent. Knock-downs in *FCLY* in Arabidopsis are hypersensitive to ABA in seed germination assays, similar to the farnesyl transferase mutant *era1* (Ziegelhoffer *et al*., 2000) and prenyl cysteine methyl esterase mutant, *icme* (Deem *et al*., 2006). The ABA hypersensitive phenotype of *fcly* plants is the result of farnesyl cysteine accumulation. This compound is an inhibitor of the ICMT enzyme (Huizinga *et al*., 2010). Plant FCLY shows much higher activity *in vitro* towards farnesyl cysteine than geranylgeranyl cysteine, which raises the possibility that geranylgeranylated proteins in plants are degraded by another, yet unknown mechanism (Crowell *et al*., 2007; Fig. 4). This seems plausible, because neighboring geranylgeranyl groups on Rab proteins may constitute a steric hindrance for the proteasome, not to mention the high hydrophobicity of such di-prenylated substrates. Whether a separate protease, specialized in hydrolysis of bulky hydrophobic side groups on prenylated substrates exists, is not known.

#### Reduction of farnesal (and geranylgeranial)

The aldehyde product of prenylated cysteine oxidation is further metabolized by the reduction of farnesal to farnesol (Crowell *et al*., 2007) by NADH-dependent farnesal dehydrogenase, FLDH (Bhandari *et al*., 2010; Fig. 4). FLDH has a wide substrate specificity but uses 15-carbon long substrates more efficiently than 20-carbon long substrates (Bhandari *et al*., 2010). *fldh* mutants in Arabidopsis are associated with an ABA-insensitive phenotype in seed germination and stomatal opening, suggesting that FLDH is a negative regulator of ABA signaling. Furthermore, expression from both genes involved in farnesylcysteine degradation in Arabidopsis, i.e. *FCLY* and *FLDH* is strongly repressed by ABA (Bhandari *et al*., 2010). A similar enzymatic activity pair involved in the recycling of prenyl groups from deprenylation of proteins was discovered in other plant species (Ahmad-Sohdi *et al*., 2015). Whether the same enzyme pair accepts geranylgeranyl cysteine as a substrate *in vivo* remains to be discovered.

Further steps of farnesol and geranylgeraniol degradation have not been described in plants, but in animals it was suggested that aldose reductase-like protein AKR1B10 serves in short isoprenoid and side chain of steroid degradation (Endo *et al*., 2009). This enzyme binds prenyl alcohol and sterol on overlapping sites, which may provide a regulatory mechanism for farnesol and geranylgeraniol versus sterol degradation (Endo *et al*., 2009).

#### Farnesol and geranylgeraniol uptake

For a long time, it has been known that plants or plant cell cultures efficiently take up farnesol and geranylgeraniol from the medium and incorporate them into compounds such as dolichols, sterols, the side chain of ubiquinone, and post-translationally modified proteins (Shipton *et al*., 1995; Thai *et al*., 1999; Wanke *et al*., 2000; Gutkowska *et al*., 2004; Huchelmann *et al*., 2016).

Although farnesyl diphosphate and geranylgeranyl diphosphate are precursors for thousands of secondary metabolism molecules and ubiquitous primary metabolism intermediates, the intracellular concentration of isoprenoid alcohol monophosphates and diphosphates is kept extremely low (Gutbrod *et al*., 2023; Romer *et al*., 2024). Farnesol is a compound that is toxic to most organisms (Gupta *et al.,* 2018). In most plants, farnesol is not well tolerated; external feeding with this compound causes bleaching of cotyledons or leaves, and later leads to necrosis (Hemmerlin and Bach, 2000; Hemmerlin, 2023; Romer *et al*., 2024), similar to up-regulation of farnesyl diphosphate synthase *FPS* overexpression (Manzano *et al.*, 2004; 2006). In non-plant organisms, farnesol in the free alcohol form is also cytotoxic (Lagace *et al*., 2002; Shirtliff *et al*., 2009; Sharma *et al*., 2023).

Free geranylgeraniol amount is also very low under standard conditions, but may be accumulated without such detrimental effects (Hemmerlin, 2023; Romer *et al*., 2024). Presumably, both farnesol and geranylgeraniol are quickly metabolized or, eventually can be stored in the form of fatty acid esters (Romer *et al*., 2024), as is known for animal cells (Verdaguer *et al*., 2022).

Farnesol and geranylgeraniol in the form of free alcohols may internally arise from dephosphorylation of newly produced FPP or GGPP by a phosphatase, possibly similar to Nudix hydrolases that release phosphate group from monoterpene phosphate (Liu *et al*., 2018) or IPP (Henry *et al*., 2018) in Arabidopsis and other plants. In humans, such specific prenyl diphosphates phosphatase activity (PDP type I) has been recently described (Elsabrouty *et al*., 2021). Furthermore, this phosphatase is engaged in geranylgeraniol release for HMGR enzyme regulation (Elsabrouty *et al*., 2021). The plant homolog for PDP type I is still missing. Prenyl aldehydes may also be released from prenylated cysteine in plants and other organisms after degradation of lipid-modified proteins by a specific lyase, and later hydrogenated to form free alcohols (Crowell *et al*., 2007; Huizinga *et al*., 2010; Fig. 4).

#### Farnesol and geranylgeraniol phosphorylation

In plants and animals, but not fungi, farnesol, and geranylgeraniol alcohols (and their corresponding aldehydes) may enter the so-called salvation pathway in which they are re-used for the synthesis of downstream compounds (Romer *et al*., 2024; Fitzpatrick *et al.,* 2011a,b; Verdaguer *et al*., 2022; Fig. 4). In this pathway, prenyl alcohol is sequentially phosphorylated first to monophosphate and later to diphosphate ester by two different enzymes, farnesol kinase and farnesyl phosphate kinase, as demonstrated using *in vitro* experiments with isolated membrane fractions from Arabidopsis cells (Fitzpatrick *et al*., 2011a). The first enzyme, farnesol kinase (FOLK) is a microsomal membrane-attached protein (but also reported from chloroplasts) catalyzing the phosphorylation of short-chain polyprenols, farnesol and geranylgeraniol, to farnesyl or geranylgeranyl phosphates, respectively (Fitzpatrick *et al*., 2011a; Romer *et al*., 2024); similar activity exists also in tobacco (Thai *et al*., 1999). The *folk* mutants in Arabidopsis grow normally in standard conditions, but under drought, 10% of flowers show homeotic transformation of organs—usually multiplication of carpel structures (Fitzpatrick *et al*., 2011a; Table 1). This phenotype, as well as inhibition of seed germination, is ABA-dependent (Fitzpatrick *et al*., 2011a, b), but may not be explained by abnormal ABA synthesis (Romer *et al*., 2024). The second phosphorylation step of farnesyl phosphate is catalyzed by a so far unspecified enzyme present in the cytoplasm. In chloroplasts an enzyme pair of similar activity was described, that are engaged in phytol recycling (vitamin E tocopherol synthesis enzymes VTE5, VTE6; Valentin *et al*., 2005; vom Dorp *et al*., 2015; Almeida *et al*., 2016). VTE5 and VTE6 accept also geranylgeraniol and geranylgeranyl phosphate as substrates. However, the enzymes which catalyze F-P, GG and GG-P phosphorylation in the cytoplasm remain to be discovered.

## Sterols: derivatives of farnesyl diphosphate

Last but not the least, sterols are derivatives of the MVA cytosolic pathway (Fig. 1). Sterol roles in cell and plant biology are beyond the scope of this article, but it should be only briefly mentioned, that these flat and rigid molecules are relatively abundant components of the biological membranes—their amount reaches several mg g^–1^ dry cell weight (Jozwiak *et al*., 2017; Lipko *et al*., 2023). Sterols have a major influence on outer membrane fluidity, permeability, and thermal phase transition (recently reviewed in Ramos-Martin and d'Amelio, 2022; for plant membranes discussed in Mamode-Cassim *et al*., 2019, summarized in Fig. 3). These unique properties of steroid molecules have a profound effect on membrane-embedded enzymes, lipid microdomains (rafts), membrane channels and signaling receptors, and influence the cell physiology, both in unicellular and multicellular organisms (Nyholm, 2015). Both in animals and plants, sterols are also a precursor for regulatory molecules: sex hormones, corticoids, and vitamin D in humans, brassinosteroids in plants. Finally, in animals, cholesterol is also a protein-modifying molecule, crucial for the correct development of body plan, e.g. cholesteroylation of the hedgehog protein (Hh) in insects and mammals is essential for Hh signal reception at receiving cells (Ciepla *et al*., 2015; Manikowski *et al*., 2021). Covalent steroylation of proteins has not been found in plants, but sterol molecules have been proposed to play an important developmental role as well (Schrick *et al*., 2014). Furthermore, sterol metabolic precursors, sterols, and oxysterols are engaged in the feedback regulation of the MVA pathway by enhancing degradation of the rate-limiting enzyme 3-hydroxy-3-methylglutaryl-CoA reductase (Fig. 1). Although different in molecular details, this mode of HMGR inhibition is adopted in all eukaryotes, including plants (Burg and Espenshade, 2011; Erffelinck and Goossens, 2018; Wangeline and Hampton, 2021).

## Dolichols: mosaic compounds of MEP and MVA pathways

Dolichols are linear isoprenoid alcohols ranging from several to up to tens of isoprenoid units (Sagami *et al*., 2018). Dolichols are synthesized from a FPP precursor which has both asymmetric double bonds in *trans*(E) configuration by consecutive additions of IPP molecules, but all double bonds in newly added isoprene units are in *cis*(Z) configuration. FPP molecule comes from the MVA-derived cytoplasmic pool of isoprenoids, but IPP may come from both MEP or MVA-derived pools, which makes dolichols quite unique mosaic compounds (Lipko *et al*., 2023; recent review on dolichol metabolism and functions in plants is provided in Gutkowska *et al*., 2025a).

Dolichol phosphate is the obligatory factor in the *N-*linked glycosylation of plant proteins with engagement of dolichol-phosphate sugar donors (Schoberer *et al*., 2018) as well as glycosylphosphatidylinositol (GPI) biosynthesis (de Coninck *et al*., 2021). The *N*-glycosylation of proteins is a key player in the quality control of protein maturation and trafficking (Saijo *et al*., 2009; Haweker *et al*., 2010). GPI-anchor provides a lipid anchor for extracellular proteins that play important roles in cell-cell recognition. Important physiological processes based on glycoprotein recognition are host-pathogen or host-symbiont interactions, plasma membrane-cell wall-extracellular matrix anchoring and gamete recognition (Varki, 2017; Strasser, 2018; de Coninck *et al*., 2021; Soni *et al*., 2024).

## Ubiquinone: an abundant, yet enigmatic molecule

In eukaryotes, ubiquinone is a central component in mitochondrial oxidative phosphorylation, mediating the electron transfer from complex I (NADH: ubiquinone oxidoreductase) and II (succinate dehydrogenase) to complex III (cytochrome bc1 oxidoreductase); it is also involved in β-oxidation of fatty acids. Ubiquinol (reduced ubiquinone) is a generalized lipid-soluble antioxidant in all cellular compartments (Xu *et al*., 2022). The ubiquinone molecule is built of a benzoquinone ring and a prenyl anchor. In plants, the dominating lipid moiety in ubiquinone are 9-isoprene units long (45 carbon) or 10-isoprene units long 50 carbon) (Xu *et al*., 2022). Ubiquinone prenyl chain is synthesized from FPP or GGPP by elongation with IPP molecules, but contrary to dolichol precursors, all the newly introduced isoprene units coming from IPP retain the double bond in *trans*(E) configuration. The IPP molecules that serve for ubiquinone side chain synthesis originate from the cytoplasmic or mitochondrial pool, but still the controversy remains on the origins of FPP and GGPP—are they autonomously synthesized by mitochondria or derived from cytoplasm (Swiezewska *et al*., 1993; Closa *et al*., 2010)? Even the localization of prenyl chain synthesis is still under dispute—does the elongating enzyme reside in the mitochondria or is the ready-to-use linear prenyl group obtained from the endoplasmic reticulum (Hirooka *et al*., 2003; Jun *et al*., 2004; Ducluzeau *et al*., 2012)?

## Conclusions and perspectives

Although MVA-derived isoprenoid molecules in plants are less abundant than chloroplast MEP-derived isoprenoids, they serve vital functions for gametophytes and sporophyte survival. Cytoplasmic FPP/GGPP metabolism is essential for plant development (embryogenesis and gametophyte function) and signaling (abscisic acid and stress) and operates through a complex, regulated network that is only partially understood. FPP and GGPP, structurally similar, and sharing a common precursor pool, show strikingly different functions in plant metabolism. The variety of FPP downstream metabolites serving in primary metabolism (sterols, ubiquinone, dolichols, farnesylated proteins) outnumber GGPP derived cytoplasmic compounds- geranylgeranylated proteins. However, GGPP biosynthetic routes and intracellular transport is much more complicated than FPP, due to the (partial) engagement of the chloroplast MEP pathway. Nevertheless, common pathways of synthesis, storage, degradation and recycling, as well as interwoven mechanisms of feedback regulation are shared by FPP and GGPP. In conclusion, the primary metabolism of short terpenoids in plants is crucial for gametophyte fertility and seed formation. In particular, protein post-translational modifications such as prenylation, glycosylation and GPI-anchoring seem to be crucial in plant developmental processes.

Future research in plant ‘prenylomics’ might be in several directions:

i) The details of the biochemical synthesis of plant MVA isoprenoids still await full description, especially taking into account that the flow of intermediates from chloroplasts has been proven. The identity of these molecules and the mechanism of their transmembrane transport into and out of chloroplast remains an open question. The degradation pathways for geranylgeranylated proteins and, most importantly, farnesol and geranylgeraniol alcohols, are not known so far in any eukaryote.

(ii) An emerging subject is also whether the short prenyl metabolism may be compartmentalized to different tissues, how and when the molecules are later transported from cell to cell as is observed in the sporophyte/gametophyte interaction or maternal sporophyte/endosperm/embryo development. A detailed description of the new mutants, combined with precise microscopy, will enable answers to these puzzling questions.

(iii) Protein substrate specificity range for each protein prenyltransferase, in the light of discovery of a new enzyme and latest findings of non-canonical substrates of already known enzymes in animals and yeast, also deserve revalidation in plants. The experimental work on many putatively prenylated proteins may lead to elucidation of novel mechanisms and functions of lipid-lipid or lipid-protein interactions, many of them universal, and many plant-specific.

(iv) Finally, the transition from computer-assisted membrane behavior modelling and biophysical experiments on model membranes enriched in prenylated peptides to experiments on real biological bilayers, with varied lipid and protein composition, lies ahead of experimental plant biologists. The expanding field of microscopic markers and biorthogonal chemistry should allow for future answers to the questions of how isoprenoids change biological membrane properties, and what their biophysical function is in the mixed lipid-protein environment.

## References

[erag069-B1] Ahmad-Sohdi NA, Seman-Kamarulzaman AF, Mohamed-Hussein ZA, Hassan M. 2015. Purification and characterization of a novel NAD(P)+-farnesol dehydrogenase from Polygonum minus leaves. PLoS One 10, e0143310. 26600471 10.1371/journal.pone.0143310 PMC4657912

[erag069-B2] Akhtar TA, Surowiecki P, Siekierska H, et al 2017. Polyprenols are synthesized by a plastidial *cis*-prenyltransferase and influence photosynthetic performance. The Plant Cell 29, 1709–1725. 28655749 10.1105/tpc.16.00796 PMC5559739

[erag069-B3] Ali F, Qanmber G, Li F, Wang Z. 2022. Updated role of ABA in seed maturation, dormancy, and germination. Journal of Advanced Research 35, 199–214. 35003801 10.1016/j.jare.2021.03.011 PMC8721241

[erag069-B4] Allorent G, Osorio S, Vu JL, et al 2015. Adjustments of embryonic photosynthetic activity modulate seed fitness in *Arabidopsis thaliana*. New Phytologist 205, 707–719. 25256557 10.1111/nph.13044

[erag069-B5] Almeida J, Azevedo Mda S, Spicher L, et al 2016. Down-regulation of tomato *PHYTOL KINASE* strongly impairs tocopherol biosynthesis and affects prenyllipid metabolism in an organ-specific manner. Journal of Experimental Botany 67, 919–934. 26596763 10.1093/jxb/erv504 PMC4737080

[erag069-B6] Amen Y, Abdelwahab G, Heraiz AA, Sallam M, Othman A. 2025. Exploring sesquiterpene lactones: structural diversity and antiviral therapeutic insights. RSC Advances 15, 1970–1988. 39845113 10.1039/d4ra08125k PMC11751675

[erag069-B7] Andrade P, Caudepon D, Altabella T, Arro M, Ferrer A, Manzano D. 2017. Complex interplays between phytosterols and plastid development. Plant Signaling & Behavior 12, e1387708. 28990832 10.1080/15592324.2017.1387708 PMC5703248

[erag069-B8] Andrews M, Huizinga DH, Crowell DN. 2010. The CaaX specificities of Arabidopsis protein prenyltransferases explain era1 and ggb phenotypes. BMC Plant Biology 10, 118. 20565889 10.1186/1471-2229-10-118 PMC3017772

[erag069-B9] Ashok S, Hildebrandt ER, Ruiz CS, Hardgrove DS, Coreno DW, Schmidt WK, Hougland JL. 2020. Protein farnesyltransferase catalyzes unanticipated farnesylation and geranylgeranylation of shortened target sequences. Biochemistry 59, 1149–1162. 32125828 10.1021/acs.biochem.0c00081 PMC7310673

[erag069-B10] Atsmon-Raz Y, Tieleman DP. 2017. Parameterization of palmitoylated cysteine, farnesylated cysteine, geranylgeranylated cysteine, and myristoylated Glycine for the Martini force field. The Journal of Physical Chemistry: B 121, 11132–11143. 29144135 10.1021/acs.jpcb.7b10175

[erag069-B11] Auger SA, Pedersen JS, Maity S, et al 2025. An alkyne-containing isoprenoid analogue based on a farnesyl diphosphate scaffold is a biologically functional universal probe for proteomic analysis. Biochemistry 64, 138–155. 39652878 10.1021/acs.biochem.4c00558 PMC11706708

[erag069-B12] Bao L, Ren J, Nguyen M, Slusarczyk AS, Thole JM, Martinez SP, Huang J, Fujita T, Running MP. 2022. The cellular function of ROP GTPase prenylation is important for multicellularity in the moss *Physcomitrium patens*. Development 149, dev200279. 35660859 10.1242/dev.200279

[erag069-B13] Barghetti A, Sjogren L, Floris M, Paredes EB, Wenkel S, Brodersen P. 2017. Heat-shock protein 40 is the key farnesylation target in meristem size control, abscisic acid signaling, and drought resistance. Genes & Development 31, 2282–2295. 29269486 10.1101/gad.301242.117 PMC5769771

[erag069-B14] Barone M, Pizzorni L, Fraaije MW, Mascotti ML, Mattevi A. 2024. Evolution, structure, and drug-metabolizing activity of mammalian prenylcysteine oxidases. The Journal of Biological Chemistry 300, 107810. 39322016 10.1016/j.jbc.2024.107810 PMC11530802

[erag069-B15] Basallo O, Perez L, Lucido A, et al 2023. Changing biosynthesis of terpenoid percursors in rice through synthetic biology. Frontiers in Plant Science 14, 1133299. 37465386 10.3389/fpls.2023.1133299 PMC10350630

[erag069-B16] Beck G, Coman D, Herren E, Ruiz-Sola MA, Rodriguez-Concepcion M, Gruissem W, Vranova E. 2013. Characterization of the GGPP synthase gene family in Arabidopsis thaliana. Plant Molecular Biology 82, 393–416. 23729351 10.1007/s11103-013-0070-z

[erag069-B17] Beck R, Sun Z, Adolf F, et al 2008. Membrane curvature induced by Arf1-GTP is essential for vesicle formation. Proceedings of the National Academy of Sciences, USA 105, 11731–11736. 10.1073/pnas.0805182105 PMC2575275 18689681

[erag069-B18] Berger BM, Kim JH, Hildebrandt ER, Davis IC, Morgan MC, Hougland JL, Schmidt WK. 2018. Protein isoprenylation in yeast targets COOH-terminal sequences not adhering to the CaaX consensus. Genetics 210, 1301–1316. 30257935 10.1534/genetics.118.301454 PMC6283164

[erag069-B19] Bergman ME, Kortbeek RWJ, Gutensohn M, Dudareva N. 2024. Plant terpenoid biosynthetic network and its multiple layers of regulation. Progress in Lipid Research 95, 101287. 38906423 10.1016/j.plipres.2024.101287

[erag069-B20] Bezeljak U, Loya H, Kaczmarek B, Saunders TE, Loose M. 2020. Stochastic activation and bistability in a rab GTPase regulatory network. Proceedings of the National Academy of Sciences, USA 117, 6540–6549. 10.1073/pnas.1921027117 PMC7104049 32161136

[erag069-B21] Bhandari J, Fitzpatrick AH, Crowell DN. 2010. Identification of a novel abscisic acid-regulated farnesol dehydrogenase from Arabidopsis. Plant Physiology 154, 1116–1127. 20807998 10.1104/pp.110.157784 PMC2971593

[erag069-B22] Bonetta D, Bayliss P, Sun S, Sage T, McCourt P. 2000. Farnesylation is involved in meristem organization in Arabidopsis. Planta 211, 182–190. 10945212 10.1007/s004250000283

[erag069-B23] Borghi M . 2025. Roles of sugar metabolism and transport in flower development. Current Opinion in Plant Biology 85, 102722. 40184919 10.1016/j.pbi.2025.102722

[erag069-B24] Bracha K, Lavy M, Yalovsky S. 2002. The Arabidopsis AtSTE24 is a CAAXProtease with broad substrate specificity. The Journal of Biological Chemistry 277, 29856–29864. 12039957 10.1074/jbc.M202916200

[erag069-B25] Bracha-Drori K, Shichrur K, Lubetzky TC, Yalovsky S. 2008. Functional analysis of Arabidopsis postprenylation CaaX processing enzymes and their function in subcellular protein targeting. Plant Physiology 148, 119–131. 18641086 10.1104/pp.108.120477 PMC2528099

[erag069-B26] Burg JS, Espenshade PJ. 2011. Regulation of HMG-CoA reductase in mammals and yeast. Progress in Lipid Research 50, 403–410. 21801748 10.1016/j.plipres.2011.07.002 PMC3184313

[erag069-B27] Cadinanos J, Varela I, Mandel DA, Schmidt WK, Diaz-Perales A, Lopez-Otin C, Freije JM. 2003. AtFACE-2, a functional prenylated protein protease from Arabidopsis thaliana related to mammalian ras-converting enzymes. The Journal of Biological Chemistry 278, 42091–42097. 12928436 10.1074/jbc.M306700200

[erag069-B28] Calero M, Chen CZ, Zhu W, Winand N, Havas KA, Gilbert PM, Burd CG, Collins RN. 2003. Dual prenylation is required for rab protein localization and function. Molecular Biology of the Cell 14, 1852–1867. 12802060 10.1091/mbc.E02-11-0707 PMC165082

[erag069-B29] Callegari S, McKinnon RA, Andrews S, de Barros Lopes MA. 2010. Atorvastatin-induced cell toxicity in yeast is linked to disruption of protein isoprenylation. FEMS Yeast Research 10, 188–198. 20002195 10.1111/j.1567-1364.2009.00593.x

[erag069-B30] Chai S, Ge FR, Feng QN, Li S, Zhang Y. 2016. *PLURIPETALA* mediates ROP2 localization and stability in parallel to *SCN1* but synergistically with *TIP1* in root hairs. The Plant Journal 86, 413–425. 27037800 10.1111/tpj.13179

[erag069-B31] Chang HY, Cheng TH, Wang AH. 2021. Structure, catalysis, and inhibition mechanism of prenyltransferase. IUBMB Life 73, 40–63. 33246356 10.1002/iub.2418 PMC7839719

[erag069-B32] Chary SN, Bultema RL, Packard CE, Crowell DN. 2002. Prenylcysteine alpha-carboxyl methyltransferase expression and function in *Arabidopsis thaliana*. The Plant Journal 32, 735–747. 12472689 10.1046/j.1365-313x.2002.01463.x

[erag069-B33] Chen CY, Balch WE. 2006. The Hsp90 chaperone complex regulates GDI-dependent rab recycling. Moleculal Biology of the Cell 17, 3494–3507. 10.1091/mbc.E05-12-1096 PMC1525227 16687576

[erag069-B34] Chevalier Q, Huchelmann A, Debie P, Mercier P, Hartmann M, Vonthron-Senecheau C, Bach TJ, Schaller H, Hemmerlin A. 2024. Methyl-jasmonate functions as a molecular switch promoting cross-talk between pathways for the biosynthesis of isoprenoid backbones used to modify proteins in plants. Plants (Basel) 13, 1110. 38674519 10.3390/plants13081110 PMC11055089

[erag069-B35] Chevalier Q, Debié P, Huchelmann A, Hemmerlin A. 2025. Protein prenylation makeovers in plants: insights into substrate diversification. International Journal of Molecular Sciences 26, 10638. 41226669 10.3390/ijms262110638 PMC12609287

[erag069-B36] Ciepla P, Magee AI, Tate EW. 2015. Cholesterylation: a tail of hedgehog. Biochemical Society Transactions 43, 262–267. 25849927 10.1042/BST20150032

[erag069-B37] Closa M, Vranova E, Bortolotti C, Bigler L, Arro M, Ferrer A, Gruissem W. 2010. The Arabidopsis thaliana FPP synthase isozymes have overlapping and specific functions in isoprenoid biosynthesis, and complete loss of FPP synthase activity causes early developmental arrest. The Plant Journal 63, 512–525. 20497375 10.1111/j.1365-313X.2010.04253.x

[erag069-B38] Coman D, Altenhoff A, Zoller S, Gruissem W, Vranová E. 2014. Distinct evolutionary strategies in the GGPPS family from plants. Frontiers in Plant Sciences 5, 230. 10.3389/fpls.2014.00230 PMC4034038 24904625

[erag069-B39] Crowell DN, Sen SE, Randall SK. 1998. Prenylcysteine alpha-carboxyl methyltransferase in suspension-cultured tobacco cells. Plant Physiology 118, 115–123. 9733531 10.1104/pp.118.1.115 PMC34848

[erag069-B40] Crowell DN, Huizinga DH, Deem AK, Trobaugh C, Denton R, Sen SE. 2007. *Arabidopsis thaliana* plants possess a specific farnesylcysteine lyase that is involved in detoxification and recycling of farnesylcysteine. The Plant Journal 50, 839–847. 17425716 10.1111/j.1365-313X.2007.03091.x

[erag069-B41] Cunillera N, Arro M, Delourme D, Karst F, Boronat A, Ferrer A. 1996. Arabidopsis thaliana contains two differentially expressed farnesyl-diphosphate synthase genes. The Journal of Biological Chemistry 271, 7774–7780. 8631820 10.1074/jbc.271.13.7774

[erag069-B42] Cunillera N, Boronat A, Ferrer A. 1997. The Arabidopsis thaliana FPS1 gene generates a novel mRNA that encodes a mitochondrial farnesyl-diphosphate synthase isoform. The Journal of Biological Chemistry 272, 15381–15388. 9182568 10.1074/jbc.272.24.15381

[erag069-B43] Cunillera N, Boronat A, Ferrer A. 2000. Spatial and temporal patterns of GUS expression directed by 5′ regions of the Arabidopsis thaliana farnesyl diphosphate synthase genes FPS1 and FPS2. Plant Molecular Biology 44, 747–758. 11202437 10.1023/a:1026588708849

[erag069-B44] Cutler S, Ghassemian M, Bonetta D, Cooney S, McCourt P. 1996. A protein farnesyl transferase involved in abscisic acid signal transduction in *Arabidopsis*. Science 273, 1239–1241. 8703061 10.1126/science.273.5279.1239

[erag069-B45] de Coninck T, Gistelinck K, Janse van Rensburg HC, Van den Ende W, Van Damme EJM. 2021. Sweet modifications modulate plant development. Biomolecules 11, 756. 34070047 10.3390/biom11050756 PMC8158104

[erag069-B46] Deem AK, Bultema RL, Crowell DN. 2006. Prenylcysteine methylesterase in Arabidopsis thaliana. Gene 380, 159–166. 16870359 10.1016/j.gene.2006.05.023

[erag069-B47] Diener AC, Li H, Zhou W, Whoriskey WJ, Nes WD, Fink GR. 2000. *STEROL METHYLTRANSFERASE 1* controls the level of cholesterol in plants. The Plant Cell 12, 853–870. 10852933 10.1105/tpc.12.6.853 PMC149089

[erag069-B48] Doll NM, Ingram GC. 2022. Embryo-endosperm interactions. Annual Review of Plant Biology 73, 293–321. 10.1146/annurev-arplant-102820-091838 35130443

[erag069-B49] Ducluzeau AL, Wamboldt Y, Elowsky CG, Mackenzie SA, Schuurink RC, Basset GJ. 2012. Gene network reconstruction identifies the authentic *trans*-prenyl diphosphate synthase that makes the solanesyl moiety of ubiquinone-9 in Arabidopsis. The Plant Journal 69, 366–375. 21950843 10.1111/j.1365-313X.2011.04796.x

[erag069-B50] Elsabrouty R, Jo Y, Hwang S, Jun DJ, DeBose-Boyd RA. 2021. Type 1 polyisoprenoid diphosphate phosphatase modulates geranylgeranyl-mediated control of HMG CoA reductase and UBIAD1. Elife 10, e64688. 34842525 10.7554/eLife.64688 PMC8641950

[erag069-B51] Endo S, Matsunaga T, Mamiya H, Ohta C, Soda M, Kitade Y, Tajima K, Zhao HT, El-Kabbani O, Hara A. 2009. Kinetic studies of AKR1B10, human aldose reductase-like protein: endogenous substrates and inhibition by steroids. Archives of Biochemistry and Biophysics 487, 1–9. 19464995 10.1016/j.abb.2009.05.009

[erag069-B52] Entova S, Guan Z, Imperiali B. 2019. Investigation of the conserved reentrant membrane helix in the monotopic phosphoglycosyl transferase superfamily supports key molecular interactions with polyprenol phosphate substrates. Archives of Biochemistry and Biophysics 675, 108111. 31563509 10.1016/j.abb.2019.108111 PMC6909930

[erag069-B53] Erffelinck ML, Goossens A. 2018. Review: endoplasmic Reticulum-associated degradation (ERAD)-dependent control of (tri)terpenoid metabolism in plants. Planta Medica 84, 874–880. 29906815 10.1055/a-0635-8369

[erag069-B54] Ferreira MJ, Silva J, Takeuchi H, Suzuki T, Higashiyama T, Coimbra S. 2024. Transcriptomic landscape of seedstick in *Arabidopsis thaliana* funiculus after fertilisation. BMC Plant Biology 24, 771. 39134964 10.1186/s12870-024-05489-4 PMC11320993

[erag069-B55] Fitzpatrick AH, Bhandari J, Crowell DN. 2011a. Farnesol kinase is involved in farnesol metabolism, ABA signaling and flower development in Arabidopsis. The Plant Journal 66, 1078–1088. 21395888 10.1111/j.1365-313X.2011.04572.x

[erag069-B56] Fitzpatrick AH, Shrestha N, Bhandari J, Crowell DN. 2011b. Roles for farnesol and ABA in Arabidopsis flower development. Plant Signaling & Behavior 6, 1189–1191. 21758018 10.4161/psb.6.8.15772 PMC3260718

[erag069-B57] Fracassi A, Marangoni M, Rosso P, Pallottini V, Fioramonti M, Siteni S, Segatto M. 2018. Statins and the brain: more than lipid lowering agents? Current Neuropharmacology 17, 59–83. 10.2174/1570159X15666170703101816 PMC6341496 28676012

[erag069-B58] Fu H, Chen Q, Yong S, Dang J, He Q, Jing D, Wu D, Liang G, Guo Q. 2025. The potential role of vesicle transport-related small GTPases rabs in abiotic stress responses. Plant Physiology and Biochemistry: PPB 219, 109411. 39729968 10.1016/j.plaphy.2024.109411

[erag069-B59] Galichet A, Gruissem W. 2003. Protein farnesylation in plants — conserved mechanisms but different targets. Current Opinion in Plant Biology 6, 530–535. 14611950 10.1016/j.pbi.2003.09.005

[erag069-B60] Galichet A, Hoyerova K, Kaminek M, Gruissem W. 2008. Farnesylation directs AtIPT3 subcellular localization and modulates cytokinin biosynthesis in Arabidopsis. Plant Physiology 146, 1155–1164. 18184738 10.1104/pp.107.107425 PMC2259095

[erag069-B61] Gao P, Xiang D, Quilichini TD, Venglat P, Pandey PK, Wang E, Gillmor CS, Datla R. 2019. Gene expression atlas of embryo development in Arabidopsis. Plant Reproduction 32, 93–104. 30762127 10.1007/s00497-019-00364-x

[erag069-B62] Gao W, Xiao S, Li HY, Tsao SW, Chye ML. 2009. *Arabidopsis thaliana* acyl-CoA-binding protein ACBP2 interacts with heavy-metal-binding farnesylated protein AtFP6. New Phytologist 181, 89–102. 18823312 10.1111/j.1469-8137.2008.02631.x

[erag069-B63] Garza RM, Tran PN, Hampton RY. 2009. Geranylgeranyl pyrophosphate is a potent regulator of HRD-dependent 3-hydroxy-3-methylglutaryl-CoA reductase degradation in yeast. The Journal of Biological Chemistry 284, 35368–35380. 19776008 10.1074/jbc.M109.023994 PMC2790966

[erag069-B64] Gerber E, Hemmerlin A, Hartmann M, et al 2009. The plastidial 2-*C*-methyl-D-erythritol 4-phosphate pathway provides the isoprenyl moiety for protein geranylgeranylation in tobacco BY-2 cells. The Plant Cell 21, 285–300. 19136647 10.1105/tpc.108.063248 PMC2648074

[erag069-B65] Gomes AQ, Ali BR, Ramalho JS, Godfrey RF, Barral DC, Hume AN, Seabra MC. 2003. Membrane targeting of rab GTPases is influenced by the prenylation motif. Molecular Biology of the Cell 14, 1882–1899. 12802062 10.1091/mbc.E02-10-0639 PMC165084

[erag069-B66] Gupta P, Sharma M, Arora N, Pruthi V, Poluri KM. 2018. Chemistry and biology of farnesol and its derivatives: quorum sensing molecules with immense therapeutic potential. Current Topics in Medical Chemistry 18(22), 1937–1954. 10.2174/1568026619666181210124159 30526460

[erag069-B67] Gupta P, Hirschberg J. 2022. The genetic components of a natural color palette: a comprehensive list of carotenoid pathway mutations in plants. Frontiers in Plant Science 12, 806184. 35069664 10.3389/fpls.2021.806184 PMC8770946

[erag069-B68] Gutbrod K, Romer J, Dormann P. 2023. Analysis of isoprenyl-phosphates by liquid chromatography-mass spectrometry. Methods in Enzymology 683, 171–190. 37087186 10.1016/bs.mie.2022.08.026

[erag069-B69] Gutkowska M, Bienkowski T, Hung VS, Wanke M, Hertel J, Danikiewicz W, Swiezewska E. 2004. Proteins are polyisoprenylated in *Arabidopsis thaliana*. Biochemical and Biophysical Research Communications 322, 998–1004. 15336563 10.1016/j.bbrc.2004.08.025

[erag069-B70] Gutkowska M, Swiezewska E. 2012. Structure, regulation and cellular functions of rab geranylgeranyl transferase and its cellular partner rab escort protein. Molecular Membrane Biology 29, 243–256. 22694141 10.3109/09687688.2012.693211

[erag069-B71] Gutkowska M, Wnuk M, Nowakowska J, Lichocka M, Stronkowski MM, Swiezewska E. 2015. Rab geranylgeranyl transferase β subunit is essential for male fertility and tip growth in Arabidopsis. Journal of Experimental Botany 66, 213–224. 25316062 10.1093/jxb/eru412 PMC4265159

[erag069-B72] Gutkowska M, Kaus-Drobek M, Hoffman-Sommer M, et al 2021. Impact of C-terminal truncations in the *Arabidopsis* rab escort protein (REP) on REP-rab interaction and plant fertility. The Plant Journal 108, 1400–1421. 34592024 10.1111/tpj.15519 PMC9293207

[erag069-B73] Gutkowska M, Swiezewska E, Szewinska J, Rojek J, Surmacz L. 2025a. Mysterious giants in the world of lipids: long linear isoprenoid functions in plant physiology and reproduction. Journal of Experimental Botany eraf481. Online ahead of print. 10.1093/jxb/eraf481 PMC13247534 41168095

[erag069-B74] Gutkowska M, Zajbt-Łuczniewska M, Buszewicz D, et al 2025b. Rab geranylgeranyl transferase activity is required for proper sterol biosynthesis in *Arabidopsis thaliana*. Plant & Cell Physiology pcaf166. Online ahead of print. 10.1093/pcp/pcaf166 PMC13078166 41369301

[erag069-B75] Hafidh S, Honys D. 2021. Reproduction multitasking: the male gametophyte. Annual Review of Plant Biology 72, 581–614. 10.1146/annurev-arplant-080620-021907 33900787

[erag069-B76] Hala M, Elias M, Zarsky V. 2005. A specific feature of the angiosperm rab escort protein (REP) and evolution of the REP/GDI superfamily. Journal of Molecular Biology 348, 1299–1313. 15854662 10.1016/j.jmb.2005.02.002

[erag069-B77] Hala M, Soukupova H, Synek L, Zarsky V. 2010. Arabidopsis RAB geranylgeranyl transferase β-subunit mutant is constitutively photomorphogenic, and has shoot growth and gravitropic defects. The Plant Journal 62, 615–627. 20180921 10.1111/j.1365-313X.2010.04172.x

[erag069-B78] Hala M, Zarsky V. 2019. Protein prenylation in plant stress responses. Molecules 24, 3906. 31671559 10.3390/molecules24213906 PMC6866125

[erag069-B79] Hartman HL, Hicks KA, Fierke CA. 2005. Peptide specificity of protein prenyltransferases is determined mainly by reactivity rather than binding affinity. Biochemistry 44, 15314–15324. 16285735 10.1021/bi0509503

[erag069-B80] Haweker H, Rips S, Koiwa H, Salomon S, Saijo Y, Chinchilla D, Robatzek S, von Schaewen A. 2010. Pattern recognition receptors require N-glycosylation to mediate plant immunity. The Journal of Biological Chemistry 285, 4629–4636. 20007973 10.1074/jbc.M109.063073 PMC2836068

[erag069-B81] Hemmerlin A . 2023. Phosphorylation of metabolites involved in salvage pathways for isoprenoid biosynthesis in plants. Kinases and Phosphatases 1, 151–166.

[erag069-B82] Hemmerlin A, Bach TJ. 2000. Farnesol-induced cell death and stimulation of 3-hydroxy-3-methylglutaryl-coenzyme A reductase activity in tobacco cv bright yellow-2 cells. Plant Physiology 123, 1257–1268. 10938345 10.1104/pp.123.4.1257 PMC59085

[erag069-B83] Hemmerlin A, Harwood JL, Bach TJ. 2012. A raison d’être for two distinct pathways in the early steps of plant isoprenoid biosynthesis? Progress in Lipid Research 51, 95–148. 22197147 10.1016/j.plipres.2011.12.001

[erag069-B84] Hemsley PA . 2020. S-acylation in plants: an expanding field. Biochemical Society Transactions 48, 529–536. 32239188 10.1042/BST20190703

[erag069-B85] Henry LK, Thomas ST, Widhalm JR, Lynch JH, Davis TC, Kessler SA, Bohlmann J, Noel JP, Dudareva N. 2018. Contribution of isopentenyl phosphate to plant terpenoid metabolism. Nature Plants 4, 721–729. 30127411 10.1038/s41477-018-0220-z

[erag069-B86] Hicks KA, Hartman HL, Fierke CA. 2005. Upstream polybasic region in peptides enhances dual specificity for prenylation by both farnesyltransferase and geranylgeranyltransferase type I. Biochemistry 44, 15325–15333. 16285736 10.1021/bi050951v

[erag069-B87] Hildebrandt ER, Arachea BT, Wiener MC, Schmidt WK. 2016. Ste24p mediates proteolysis of both isoprenylated and non-prenylated oligopeptides. The Journal of Biological Chemistry 291, 14185–14198. 27129777 10.1074/jbc.M116.718197 PMC4933176

[erag069-B88] Hirooka K, Bamba T, Fukusaki E, Kobayashi A. 2003. Cloning and kinetic characterization of Arabidopsis thaliana solanesyl diphosphate synthase. The Biochemical Journal 370, 679–686. 12437513 10.1042/BJ20021311 PMC1223189

[erag069-B89] Houten SM, Schneiders MS, Wanders RJ, Waterham HR. 2003. Regulation of isoprenoid/cholesterol biosynthesis in cells from mevalonate kinase-deficient patients. The Journal of Biological Chemistry 278, 5736–5743. 12477733 10.1074/jbc.M206564200

[erag069-B90] Huchelmann A, Brahim MS, Gerber E, Tritsch D, Bach TJ, Hemmerlin A. 2016. Farnesol-mediated shift in the metabolic origin of prenyl groups used for protein prenylation in plants. Biochimie 127, 95–102. 27138105 10.1016/j.biochi.2016.04.021

[erag069-B91] Huizinga DH, Omosegbon O, Omery B, Crowell DN. 2008. Isoprenylcysteine methylation and demethylation regulate abscisic acid signaling in *Arabidopsis*. The Plant Cell 20, 2714–2728. 18957507 10.1105/tpc.107.053389 PMC2590716

[erag069-B92] Huizinga DH, Denton R, Koehler KG, Tomasello A, Wood L, Sen SE, Crowell DN. 2010. Farnesylcysteine lyase is involved in negative regulation of abscisic acid signaling in Arabidopsis. Molecular Plant 3, 143–155. 19969520 10.1093/mp/ssp091 PMC2807925

[erag069-B93] Ishiguro S, Nishimori Y, Yamada M, Saito H, Suzuki T, Nakagawa T, Miyake H, Okada K, Nakamura K. 2010. The Arabidopsis FLAKY POLLEN1 gene encodes a 3-hydroxy-3-methylglutaryl-coenzyme A synthase required for development of tapetum-specific organelles and fertility of pollen grains. Plant & Cell Physiology 51, 896–911. 20484369 10.1093/pcp/pcq068

[erag069-B94] Jang H, Abraham SJ, Chavan TS, Hitchinson B, Khavrutskii L, Tarasova NI, Nussinov R, Gaponenko V. 2015. Mechanisms of membrane binding of small GTPase K-Ras4B farnesylated hypervariable region. The Journal of Biological Chemistry 290, 9465–9477. 25713064 10.1074/jbc.M114.620724 PMC4392252

[erag069-B95] Janosi L, Gorfe AA. 2010. Segregation of negatively charged phospholipids by the polycationic and farnesylated membrane anchor of kras. Biophysical Journal 99, 3666–3674. 21112291 10.1016/j.bpj.2010.10.031 PMC2998625

[erag069-B96] Janosi L, Li Z, Hancock JF, Gorfe AA. 2012. Organization, dynamics, and segregation of ras nanoclusters in membrane domains. Proceedings of the National Academy of Sciences, USA 109, 8097–8102. 10.1073/pnas.1200773109 PMC3361399 22562795

[erag069-B97] Johnson CD, Chary SN, Chernoff EA, Zeng Q, Running MP, Crowell DN. 2005. Protein geranylgeranyltransferase I is involved in specific aspects of abscisic acid and auxin signaling in Arabidopsis. Plant Physiology 139, 722–733. 16183844 10.1104/pp.105.065045 PMC1255991

[erag069-B98] Jozwiak A, Gutkowska M, Gawarecka K, Surmacz L, Buczkowska A, Lichocka M, Nowakowska J, Swiezewska E. 2015. POLYPRENOL REDUCTASE2 deficiency is lethal in Arabidopsis due to male sterility. The Plant Cell 27, 3336–3353. 26628744 10.1105/tpc.15.00463 PMC4707453

[erag069-B99] Jozwiak A, Lipko A, Kania M, et al 2017. Modeling of dolichol mass Spectra isotopic envelopes as a tool to monitor isoprenoid biosynthesis. Plant Physiology 174, 857–874. 28385729 10.1104/pp.17.00036 PMC5462023

[erag069-B100] Jun L, Saiki R, Tatsumi K, Nakagawa T, Kawamukai M. 2004. Identification and subcellular localization of two solanesyl diphosphate synthases from Arabidopsis thaliana. Plant & Cell Physiology 45, 1882–1888. 15653808 10.1093/pcp/pch211

[erag069-B101] Justyna K, Das R, Lorimer EL, et al 2023. Synthesis, enzymatic peptide incorporation, and applications of diazirine-containing isoprenoid diphosphate analogues. Organic Letters 25, 6767–6772. 37669435 10.1021/acs.orglett.3c02736 PMC10755972

[erag069-B102] Keim V, Manzano D, Fernandez FJ, Closa M, Andrade P, Caudepon D, Bortolotti C, Vega MC, Arro M, Ferrer A. 2012. Characterization of Arabidopsis FPS isozymes and FPS gene expression analysis provide insight into the biosynthesis of isoprenoid precursors in seeds. PLoS One 7, e49109. 23145086 10.1371/journal.pone.0049109 PMC3492304

[erag069-B103] Kirsten ML, Baron RA, Seabra MC, Ces O. 2013. Rab1a and Rab5a preferentially bind to binary lipid compositions with higher stored curvature elastic energy. Molecular Membrane Biology 30, 303–314. 23815289 10.3109/09687688.2013.818725

[erag069-B104] Kobayashi K, Suzuki M, Muranaka T, Nagata N. 2018. The mevalonate pathway but not the methylerythritol phosphate pathway is critical for elaioplast and pollen coat development in Arabidopsis. Plant Biotechnology 35, 381–385. 31892826 10.5511/plantbiotechnology.18.0702a PMC6905213

[erag069-B105] Kopcsayova D, Vranova E. 2019. Functional gene network of prenyltransferases in Arabidopsis thaliana. Molecules 24, 4556. 31842481 10.3390/molecules24244556 PMC6943727

[erag069-B106] Koukos PI, Dehghani-Ghahnaviyeh S, Velez-Vega C, Manchester J, Tieleman DP, Duca JS, Souza PCT, Cournia Z. 2023. Martini 3 force field parameters for protein lipidation post-translational modifications. Journal of Chemical Theory and Computation 19, 8901–8918. 38019969 10.1021/acs.jctc.3c00604

[erag069-B107] Krzysiak AJ, Aditya AV, Hougland JL, Fierke CA, Gibbs RA. 2010. Synthesis and screening of a CaaL peptide library versus FTase reveals a surprising number of substrates. Bioorganic & Medicinal Chemistry Letters 20, 767–770. 20005705 10.1016/j.bmcl.2009.11.011 PMC2922960

[erag069-B108] Kuchay S, Wang H, Marzio A, Jain K, Homer H, Fehrenbacher N, Philips MR, Zheng N, Pagano M. 2019. GGTase3 is a newly identified geranylgeranyltransferase targeting a ubiquitin ligase. Nature Structural & Molecular Biology 26, 628–636. 10.1038/s41594-019-0249-3 PMC6609460 31209342

[erag069-B109] Kulakowski G, Bousquet H, Manneville JB, Bassereau P, Goud B, Oesterlin LK. 2018. Lipid packing defects and membrane charge control RAB GTPase recruitment. Traffic 19, 536–545. 29573133 10.1111/tra.12568 PMC6032855

[erag069-B110] Lafon-Placette C, Köhler C. 2014. Embryo and endosperm, partners in seed development. Current Opinion in Plant Biology 17, 64–69. 24507496 10.1016/j.pbi.2013.11.008

[erag069-B111] Lagace TA, Miller JR, Ridgway ND. 2002. Caspase processing and nuclear export of CTP:phosphocholine cytidylyltransferase alpha during farnesol-induced apoptosis. Molecular and Cellular Biology 22, 4851–4862. 12052891 10.1128/MCB.22.13.4851-4862.2002 PMC133913

[erag069-B112] Lan P, Li W, Wang H, Ma W. 2010. Characterization, sub-cellular localization and expression profiling of the isoprenylcysteine methylesterase gene family in Arabidopsis thaliana. BMC Plant Biology 10, 212. 20868530 10.1186/1471-2229-10-212 PMC3017835

[erag069-B113] Larsson E, Vivian-Smith A, Offringa R, Sundberg E. 2017. Auxin homeostasis in Arabidopsis ovules is anther-dependent at maturation and changes dynamically upon fertilization. Frontiers in Plant Science 8, 1735. 29067034 10.3389/fpls.2017.01735 PMC5641375

[erag069-B114] Lee M, Wickner W, Song H. 2020. A rab prenyl membrane-anchor allows effector recognition to be regulated by guanine nucleotide. Proceedings of the National Academy of Sciences, USA 117, 7739–7744. 10.1073/pnas.2000923117 PMC7148575 32213587

[erag069-B115] Leichner GS, Avner R, Harats D, Roitelman J. 2011. Metabolically regulated endoplasmic reticulum-associated degradation of 3-hydroxy-3-methylglutaryl-CoA reductase: evidence for requirement of a geranylgeranylated protein. The Journal of Biological Chemistry 286, 32150–32161. 21778231 10.1074/jbc.M111.278036 PMC3173168

[erag069-B116] Lemichez E, Wu Y, Sanchez JP, Mettouchi A, Mathur J, Chua NH. 2001. Inactivation of AtRac1 by abscisic acid is essential for stomatal closure. Genes & Development 15, 1808–1816. 11459830 10.1101/gad.900401 PMC312738

[erag069-B117] Leung KF, Baron R, Ali BR, Magee AI, Seabra MC. 2007. Rab GTPases containing a CAAX motif are processed post-geranylgeranylation by proteolysis and methylation. The Journal of Biological Chemistry 282, 1487–1497. 17114793 10.1074/jbc.M605557200

[erag069-B118] Levental I, Grzybek M, Simons K. 2010. Greasing their way: lipid modifications determine protein association with membrane rafts. Biochemistry 49, 6305–6316. 20583817 10.1021/bi100882y

[erag069-B119] Li Z, Janosi L, Gorfe AA. 2012. Formation and domain partitioning of H-ras peptide nanoclusters: effects of peptide concentration and lipid composition. Journal of the American Chemical Society 134, 17278–17285. 22994893 10.1021/ja307716z PMC3479155

[erag069-B120] Liebers M, Grubler B, Chevalier F, Lerbs-Mache S, Merendino L, Blanvillain R, Pfannschmidt T. 2017. Regulatory shifts in plastid transcription play a key role in morphological conversions of plastids during plant development. Frontiers in Plant Science 8, 23. 28154576 10.3389/fpls.2017.00023 PMC5243808

[erag069-B121] Lima RB, Figueiredo DD. 2024. Sex on steroids: how brassinosteroids shape reproductive development in flowering plants. Plant & Cell Physiology 65, 1581–1600. 38668644 10.1093/pcp/pcae050 PMC11558549

[erag069-B122] Lindner H, Kessler SA, Müller LM, Shimosato-Asano H, Boisson-Dernier A, Grossniklaus U. 2015. TURAN and EVAN mediate pollen tube reception in Arabidopsis synergids through protein glycosylation. PLoS Biology 13, e1002139. 25919390 10.1371/journal.pbio.1002139 PMC4412406

[erag069-B123] Lipko A, Paczkowski C, Perez-Fons L, Fraser PD, Kania M, Hoffman-Sommer M, Danikiewicz W, Rohmer M, Poznanski J, Swiezewska E. 2023. Divergent contribution of the MVA and MEP pathways to the formation of polyprenols and dolichols in Arabidopsis. The Biochemical Journal 480, 495–520. 37022297 10.1042/BCJ20220578 PMC10212524

[erag069-B124] Liscum L . 2011. Chapter 14: Cholesterol biosynthesis. In: Vance DE, Vance JE, eds. Biochemistry of lipids, lipoproteins and membranes (5th edn.). Elsevier, 399–421.

[erag069-B125] Liu J, Guan Z, Liu H, Qi L, Zhang D, Zou T, Yin P. 2018. Structural insights into the substrate recognition mechanism of Arabidopsis GPP-bound NUDX1 for noncanonical monoterpene biosynthesis. Molecular Plant 11, 218–221. 29066356 10.1016/j.molp.2017.10.006

[erag069-B126] Lu JY, Hofmann SL. 2006. Thematic review series: lipid posttranslational modifications. Lysosomal metabolism of lipid-modified proteins. Journal of Lipid Research 47, 1352–1357. 16627894 10.1194/jlr.R600010-JLR200

[erag069-B127] Ma YT, Gilbert BA, Rando RR. 1995. Farnesylcysteine analogs to probe role of prenylated protein methyltransferase. Methods in Enzymology 250, 226–234. 7651154 10.1016/0076-6879(95)50075-8

[erag069-B128] Mamode-Cassim A, Gouguet P, Gronnier J, Laurent N, Germain V, Grison M, Boutte Y, Gerbeau-Pissot P, Simon-Plas F, Mongrand S. 2019. Plant lipids: key players of plasma membrane organization and function. Progress in Lipid Research 73, 1–27. 30465788 10.1016/j.plipres.2018.11.002

[erag069-B129] Manikowski D, Ehring K, Gude F, Jakobs P, Froese J, Grobe K. 2021. Hedgehog lipids: promotors of alternative morphogen release and signaling? : conflicting findings on lipidated hedgehog transport and signaling can be explained by alternative regulated mechanisms to release the morphogen. BioEssays: News and Reviews in Molecular, Cellular and Developmental Biology 43, e2100133. 34611914 10.1002/bies.202100133

[erag069-B130] Manzano D, Fernandez-Busquets X, Schaller H, Gonzalez V, Boronat A, Arro M, Ferrer A. 2004. The metabolic imbalance underlying lesion formation in Arabidopsis thaliana overexpressing farnesyl diphosphate synthase (isoform 1S) leads to oxidative stress and is triggered by the developmental decline of endogenous HMGR activity. Planta 219, 982–992. 15605175 10.1007/s00425-004-1301-y

[erag069-B131] Manzano D, Busquets A, Closa M, Hoyerova K, Schaller H, Kaminek M, Arro M, Ferrer A. 2006. Overexpression of farnesyl diphosphate synthase in Arabidopsis mitochondria triggers light-dependent lesion formation and alters cytokinin homeostasis. Plant Molecular Biology 61, 195–213. 16786301 10.1007/s11103-006-6263-y

[erag069-B132] Manzano D, Andrade P, Caudepon D, Altabella T, Arro M, Ferrer A. 2016. Suppressing farnesyl diphosphate synthase alters chloroplast development and triggers sterol-dependent induction of jasmonate- and fe-related responses. Plant Physiology 172, 93–117. 27382138 10.1104/pp.16.00431 PMC5074618

[erag069-B133] Marchwicka A, Kaminska D, Monirialamdari M, Blazewska KM, Gendaszewska-Darmach E. 2022. Protein prenyltransferases and their inhibitors: structural and functional characterization. International Journal of Molecular Sciences 23, 5424. 35628237 10.3390/ijms23105424 PMC9141697

[erag069-B134] Martiniere A, Moreau P. 2020. Complex roles of rabs and SNAREs in the secretory pathway and plant development: a never-ending story. Journal of Microscopy 280, 140–157. 32761815 10.1111/jmi.12952

[erag069-B135] Masferrer A, Arro M, Manzano D, Schaller H, Fernandez-Busquets X, Moncalean P, Fernandez B, Cunillera N, Boronat A, Ferrer A. 2002. Overexpression of *Arabidopsis thaliana* farnesyl diphosphate synthase (FPS1S) in transgenic *Arabidopsis* induces a cell death/senescence-like response and reduced cytokinin levels. The Plant Journal 30, 123–132. 12000449 10.1046/j.1365-313x.2002.01273.x

[erag069-B136] Maxwell KN, Zhou Y, Hancock JF. 2018. Rac1 nanoscale organization on the plasma membrane is driven by lipid binding specificity encoded in the membrane anchor. Molecular and Cellular Biology 38, e00186-18. 29967243 10.1128/MCB.00186-18 PMC6113602

[erag069-B137] Michaelson D, Ali W, Chiu VK, Bergo M, Silletti J, Wright L, Young SG, Philips M. 2005. Postprenylation *CAAX* processing is required for proper localization of ras but not rho GTPases. Molecular Biology of the Cell 16, 1606–1616. 15659645 10.1091/mbc.E04-11-0960 PMC1073645

[erag069-B138] Moreira G, Ferreira MEP, Linhares FS. 2025. Identity transitions of tapetum phases: insights into vesicular dynamics and in mortem support during pollen maturation. Plants (Basel) 14, 749. 40094707 10.3390/plants14050749 PMC11902102

[erag069-B139] Nagel R, Bernholz C, Vranova E, Kosuth J, Bergau N, Ludwig S, Wessjohann L, Gershenzon J, Tissier A, Schmidt A. 2015. *Arabidopsis thaliana* isoprenyl diphosphate synthases produce the C_25_ intermediate geranylfarnesyl diphosphate. The Plant Journal 84, 847–859. 26505977 10.1111/tpj.13064

[erag069-B140] Nielsen E . 2020. The small GTPase superfamily in plants: a conserved regulatory module with novel functions. Annual Review of Plant Biology 71, 247–272. 10.1146/annurev-arplant-112619-025827 32442390

[erag069-B141] Northey JG, Liang S, Jamshed M, Deb S, Foo E, Reid JB, McCourt P, Samuel MA. 2016. Farnesylation mediates brassinosteroid biosynthesis to regulate abscisic acid responses. Nature Plants 2, 16114. 27455172 10.1038/nplants.2016.114

[erag069-B142] Nyholm TK . 2015. Lipid-protein interplay and lateral organization in biomembranes. Chemistry and Physics of Lipids 189, 48–55. 26036778 10.1016/j.chemphyslip.2015.05.008

[erag069-B143] O'Brien M, Chantha SC, Rahier A, Matton DP. 2005. Lipid signaling in plants. Cloning and expression analysis of the obtusifoliol 14α-demethylase from *Solanum chacoense* bitt., a pollination- and fertilization-induced gene with both obtusifoliol and lanosterol demethylase activity. Plant Physiology 139, 734–749. 16169959 10.1104/pp.105.066639 PMC1255992

[erag069-B144] Ogata T, Nagatoshi Y, Yamagishi N, Yoshikawa N, Fujita Y. 2017. Virus-induced down-regulation of GmERA1A and GmERA1B genes enhances the stomatal response to abscisic acid and drought resistance in soybean. PLoS One 12, e0175650. 28419130 10.1371/journal.pone.0175650 PMC5395220

[erag069-B145] Ogata T, Ishizaki T, Fujita M, Fujita Y. 2020. CRISPR/Cas9-targeted mutagenesis of OsERA1 confers enhanced responses to abscisic acid and drought stress and increased primary root growth under nonstressed conditions in rice. PLoS One 15, e0243376. 33270810 10.1371/journal.pone.0243376 PMC7714338

[erag069-B146] Ohnuma S, Nakazawa T, Hemmi H, Hallberg AM, Koyama T, Ogura K, Nishino T. 1996. Conversion from farnesyl diphosphate synthase to geranylgeranyl diphosphate synthase by random chemical mutagenesis. The Journal of Biological Chemistry 271, 10087–10095. 8626566 10.1074/jbc.271.17.10087

[erag069-B147] Okada K, Saito T, Nakagawa T, Kawamukai M, Kamiya Y. 2000. Five geranylgeranyl diphosphate synthases expressed in different organs are localized into three subcellular compartments in Arabidopsis. Plant Physiology 122, 1045–1056. 10759500 10.1104/pp.122.4.1045 PMC58939

[erag069-B148] Okada K, Ohara K, Yazaki K, Nozaki K, Uchida N, Kawamukai M, Nojiri H, Yamane H. 2004. The AtPPT1 gene encoding 4-hydroxybenzoate polyprenyl diphosphate transferase in ubiquinone biosynthesis is required for embryo development in *Arabidopsis thaliana*. Plant Molecular Biology 55, 567–577. 15604701 10.1007/s11103-004-1298-4

[erag069-B149] Okada K, Kasahara H, Yamaguchi S, Kawaide H, Kamiya Y, Nojiri H, Yamane H. 2008. Genetic evidence for the role of isopentenyl diphosphate isomerases in the mevalonate pathway and plant development in Arabidopsis. Plant & Cell Physiology 49, 604–616. 18303110 10.1093/pcp/pcn032

[erag069-B150] Petrova M, Miladinova-Georgieva K, Geneva M. 2024. Influence of abiotic and biotic elicitors on organogenesis, biomass accumulation, and production of key secondary metabolites in Asteraceae plants. International Journal of Molecular Sciences 25, 4197. 38673783 10.3390/ijms25084197 PMC11050642

[erag069-B151] Plackett ARG, Wilson ZA. 2017. Gibberellins and plant reproduction. In: Roberts JA, ed. Annual plant reviews, vol. 49 The Gibberellins. Wiley, online, 10.1002/9781119312994.apr0540

[erag069-B152] Pokhilko A, Bou-Torrent J, Pulido P, Rodriguez-Concepcion M, Ebenhoh O. 2015. Mathematical modelling of the diurnal regulation of the MEP pathway in *Arabidopsis*. New Phytologist 206, 1075–1085. 25598499 10.1111/nph.13258

[erag069-B153] Povilus RA, Gehring M. 2022. Maternal-filial transfer structures in endosperm: a nexus of nutritional dynamics and seed development. Current Opinion in Plant Biology 65, 102121. 34801784 10.1016/j.pbi.2021.102121

[erag069-B154] Prodromou C . 2023. An editorial on the special issue ‘Hsp90 structure, mechanism and disease’. Biomolecules 13, 547. 36979482 10.3390/biom13030547 PMC10045984

[erag069-B155] Pu X, Dong X, Li Q, Chen Z, Liu L. 2021. An update on the function and regulation of methylerythritol phosphate and mevalonate pathways and their evolutionary dynamics. Journal of Integrative Plant Biology 63, 1211–1226. 33538411 10.1111/jipb.13076

[erag069-B156] Pulido P, Perello C, Rodriguez-Concepcion M. 2012. New insights into plant isoprenoid metabolism. Molecular Plant 5, 964–967. 22972017 10.1093/mp/sss088

[erag069-B157] Pullen M, Clark N, Zarinkamar F, Topping J, Lindsey K. 2010. Analysis of vascular development in the hydra sterol biosynthetic mutants of Arabidopsis. PLoS One 5, e12227. 20808926 10.1371/journal.pone.0012227 PMC2923191

[erag069-B158] Qiao Z, Hu H, Shi S, Yuan X, Yan B, Chen L. 2021. An update on the function, biosynthesis and regulation of floral volatile terpenoids. Horticulturae 7, 451.

[erag069-B159] Qin X, Li W, Liu Y, Tan M, Ganal M, Chetelat RT. 2018. A farnesyl pyrophosphate synthase gene expressed in pollen functions in *S*-RNase-independent unilateral incompatibility. The Plant Journal 93, 417–430. 29206320 10.1111/tpj.13796

[erag069-B160] Qin X, Ng S, Paul E, Chetelat RT. 2025. Genetic interactions and natural variation underlying S-RNase-independent unilateral incompatibility in *Solanum*. The Plant Journal 123, e70412. 40839807 10.1111/tpj.70412

[erag069-B161] Ramos-Martin F, D'Amelio N. 2022. Biomembrane lipids: when physics and chemistry join to shape biological activity. Biochimie 203, 118–138. 35926681 10.1016/j.biochi.2022.07.011

[erag069-B162] Reid TS, Terry KL, Casey PJ, Beese LS. 2004. Crystallographic analysis of CaaX prenyltransferases complexed with substrates defines rules of protein substrate selectivity. Journal of Molecular Biology 343, 417–433. 15451670 10.1016/j.jmb.2004.08.056

[erag069-B163] Robert HS . 2019. Molecular communication for coordinated seed and fruit development: what can we learn from auxin and sugars? International Journal of Molecular Sciences 20, 936. 30795528 10.3390/ijms20040936 PMC6412287

[erag069-B164] Rodriguez-Concepcion M, Yalovsky S, Zik M, Fromm H, Gruissem W. 1999. The prenylation status of a novel plant calmodulin directs plasma membrane or nuclear localization of the protein. The EMBO Journal 18, 1996–2007. 10202162 10.1093/emboj/18.7.1996 PMC1171284

[erag069-B165] Rodriguez-Concepcion M, Toledo-Ortiz G, Yalovsky S, Caldelari D, Gruissem W. 2000. Carboxyl-methylation of prenylated calmodulin CaM53 is required for efficient plasma membrane targeting of the protein. The Plant Journal 24, 775–784. 11135111 10.1046/j.1365-313x.2000.00924.x

[erag069-B166] Rodriguez-Concepcion M, Fores O, Martinez-Garcia JF, Gonzalez V, Phillips MA, Ferrer A, Boronat A. 2004. Distinct light-mediated pathways regulate the biosynthesis and exchange of isoprenoid precursors during Arabidopsis seedling development. The Plant Cell 16, 144–156. 14660801 10.1105/tpc.016204 PMC301401

[erag069-B167] Rodriguez-Concepcion M, Boronat A. 2015. Breaking new ground in the regulation of the early steps of plant isoprenoid biosynthesis. Current Opinion in Plant Biology 25, 17–22. 25909859 10.1016/j.pbi.2015.04.001

[erag069-B168] Rojek J, Tucker MR, Pinto SC, Rychlowski M, Lichocka M, Soukupova H, Nowakowska J, Bohdanowicz J, Surmacz G, Gutkowska M. 2021a. Rab-dependent vesicular traffic affects female gametophyte development in Arabidopsis. Journal of Experimental Botany 72, 320–340. 32939545 10.1093/jxb/eraa430 PMC7853608

[erag069-B169] Rojek J, Tucker MR, Rychlowski M, Nowakowska J, Gutkowska M. 2021b. The rab geranylgeranyl transferase Beta subunit is essential for embryo and seed development in Arabidopsis thaliana. International Journal of Molecular Sciences 22, 7907. 34360673 10.3390/ijms22157907 PMC8347404

[erag069-B170] Romer J, Gutbrod K, Schuppener A, Melzer M, Muller-Schussele SJ, Meyer AJ, Dormann P. 2024. Tocopherol and phylloquinone biosynthesis in chloroplasts requires the phytol kinase VITAMIN E PATHWAY GENE5 (VTE5) and the farnesol kinase (FOLK). The Plant Cell 36, 1140–1158. 38124486 10.1093/plcell/koad316 PMC10980339

[erag069-B171] Ruiz-Sola MA, Barja MV, Manzano D, Llorente B, Schipper B, Beekwilder J, Rodriguez-Concepcion M. 2016a. A single Arabidopsis gene encodes two differentially targeted geranylgeranyl diphosphate synthase isoforms. Plant Physiology 172, 1393–1402. 27707890 10.1104/pp.16.01392 PMC5100792

[erag069-B172] Ruiz-Sola MA, Coman D, Beck G, et al 2016b. *Arabidopsis* GERANYLGERANYL DIPHOSPHATE SYNTHASE 11 is a hub isozyme required for the production of most photosynthesis-related isoprenoids. New Phytologist 209, 252–264. 26224411 10.1111/nph.13580

[erag069-B173] Running MP, Fletcher JC, Meyerowitz EM. 1998. The *WIGGUM* gene is required for proper regulation of floral meristem size in *Arabidopsis*. Development 125, 2545–2553. 9636070 10.1242/dev.125.14.2545

[erag069-B174] Running MP, Lavy M, Sternberg H, Galichet A, Gruissem W, Hake S, Ori N, Yalovsky S. 2004. Enlarged meristems and delayed growth in *plp* mutants result from lack of CaaX prenyltransferases. Proceedings of the National Academy of Sciences, USA 101, 7815–7820. 10.1073/pnas.0402385101 PMC419689 15128936

[erag069-B175] Ruppel NJ, Kropp KN, Davis PA, Martin AE, Luesse DR, Hangarter RP. 2013. Mutations in *GERANYLGERANYL DIPHOSPHATE SYNTHASE 1* affect chloroplast development in Arabidopsis thaliana (Brassicaceae). American Journal of Botany 100, 2074–2084. 24081146 10.3732/ajb.1300124

[erag069-B176] Sagami H, Swiezewska E, Shidoji Y. 2018. The history and recent advances in research of polyprenol and its derivatives. Bioscience, Biotechnology, and Biochemistry 82, 947–955. 29297247 10.1080/09168451.2017.1411775

[erag069-B177] Saijo Y, Tintor N, Lu X, Rauf P, Pajerowska-Mukhtar K, Haweker H, Dong X, Robatzek S, Schulze-Lefert P. 2009. Receptor quality control in the endoplasmic reticulum for plant innate immunity. The EMBO Journal 28, 3439–3449. 19763087 10.1038/emboj.2009.263 PMC2776098

[erag069-B178] Sakata N, Shirakawa R, Goto K, Trinh DA, Horiuchi H. 2021. Double prenylation of SNARE protein Ykt6 is required for lysosomal hydrolase trafficking. Journal of Biochemistry 169, 363–370. 33035318 10.1093/jb/mvaa111

[erag069-B179] Schey GL, Hildebrandt ER, Wang Y, et al 2024. Library screening, in vivo confirmation, and structural and bioinformatic analysis of pentapeptide sequences as substrates for protein farnesyltransferase. International Journal of Molecular Sciences 25, 5324. 38791363 10.3390/ijms25105324 PMC11121372

[erag069-B180] Schoberer J, Shin YJ, Vavra U, Veit C, Strasser R. 2018. Analysis of protein glycosylation in the ER. Methods in Molecular Biology 1691, 205–222. 29043680 10.1007/978-1-4939-7389-7_16 PMC7039702

[erag069-B181] Schrick K, Bruno M, Khosla A, et al 2014. Shared functions of plant and mammalian StAR-related lipid transfer (START) domains in modulating transcription factor activity. BMC Biology 12, 70. 25159688 10.1186/s12915-014-0070-8 PMC4169639

[erag069-B182] Schumacher MM, Elsabrouty R, Seemann J, Jo Y, DeBose-Boyd RA. 2015. The prenyltransferase UBIAD1 is the target of geranylgeraniol in degradation of HMG CoA reductase. elife 4, e05560. 25742604 10.7554/eLife.05560 PMC4374513

[erag069-B183] Sela A, Piskurewicz U, Megies C, Mene-Saffrane L, Finazzi G, Lopez-Molina L. 2020. Embryonic photosynthesis affects post-germination plant growth. Plant Physiology 182, 2166–2181. 32060052 10.1104/pp.20.00043 PMC7140907

[erag069-B184] Shahinian S, Silvius JR. 1995. Doubly-lipid-modified protein sequence motifs exhibit long-lived anchorage to lipid bilayer membranes. Biochemistry 34, 3813–3822. 7893678 10.1021/bi00011a039

[erag069-B185] Sharma H, Sehgal R, Shekhar N, Shoeran G, Kaur U, Medhi B. 2023. Antiparasitic effect of farnesol against leishmania major: a rationale from in vitro and in silico investigations. PLoS One 18, e0293290. 37930969 10.1371/journal.pone.0293290 PMC10627473

[erag069-B186] Shi W, Zeng Q, Kunkel BN, Running MP. 2016. Arabidopsis rab geranylgeranyltransferases demonstrate redundancy and broad substrate specificity in vitro. The Journal of Biological Chemistry 291, 1398–1410. 26589801 10.1074/jbc.M115.673491 PMC4714223

[erag069-B187] Shipton CA, Parmryd I, Swiezewska E, Andersson B, Dallner G. 1995. Isoprenylation of plant proteins in vivo. Isoprenylated proteins are abundant in the mitochondria and nuclei of spinach. The Journal of Biological Chemistry 270, 566–572. 7822281 10.1074/jbc.270.2.566

[erag069-B188] Shirakawa R, Goto-Ito S, Goto K, et al 2020. A SNARE geranylgeranyltransferase essential for the organization of the Golgi apparatus. The EMBO Journal 39, e104120. 32128853 10.15252/embj.2019104120 PMC7156963

[erag069-B189] Shirtliff ME, Krom BP, Meijering RA, Peters BM, Zhu J, Scheper MA, Harris ML, Jabra-Rizk MA. 2009. Farnesol-induced apoptosis in *Candida albicans*. Antimicrobial Agents and Chemotherapy 53, 2392–2401. 19364863 10.1128/AAC.01551-08 PMC2687256

[erag069-B190] Silvius JR, l'Heureux F. 1994. Fluorimetric evaluation of the affinities of isoprenylated peptides for lipid bilayers. Biochemistry 33, 3014–3022. 8130214 10.1021/bi00176a034

[erag069-B191] Sjogren L, Floris M, Barghetti A, Vollmy F, Linding R, Brodersen P. 2018. Farnesylated heat shock protein 40 is a component of membrane-bound RISC in Arabidopsis. The Journal of Biological Chemistry 293, 16608–16622. 30194279 10.1074/jbc.RA118.003887 PMC6204899

[erag069-B192] Skorupinska-Tudek K, Poznanski J, Wojcik J, et al 2008. Contribution of the mevalonate and methylerythritol phosphate pathways to the biosynthesis of dolichols in plants. The Journal of Biological Chemistry 283, 21024–21035. 18502754 10.1074/jbc.M706069200 PMC3258935

[erag069-B193] Song J, Sun S, Ren H, Grison M, Boutte Y, Bai W, Men S. 2019. The SMO1 family of sterol 4α-methyl oxidases is essential for auxin- and cytokinin-regulated embryogenesis. Plant Physiology 181, 578–594. 31341004 10.1104/pp.19.00144 PMC6776873

[erag069-B194] Soni KK, Gurjar K, Ranjan A, Sinha S, Srivastava M, Verma V. 2024. Post-translational modifications control the signal at the crossroads of plant-pathogen interactions. Journal of Experimental Botany 75, 6957–6979. 39177255 10.1093/jxb/erae358

[erag069-B195] Sorek N, Gutman O, Bar E, et al 2011. Differential effects of prenylation and *S*-acylation on type I and II ROPS membrane interaction and function. Plant Physiology 155, 706–720. 21139084 10.1104/pp.110.166850 PMC3032461

[erag069-B196] Stein V, Kubala MH, Steen J, Grimmond SM, Alexandrov K. 2015. Towards the systematic mapping and engineering of the protein prenylation machinery in Saccharomyces cerevisiae. PLoS One 10, e0120716. 25768003 10.1371/journal.pone.0120716 PMC4358939

[erag069-B197] Storck EM, Morales-Sanfrutos J, Serwa RA, et al 2019. Dual chemical probes enable quantitative system-wide analysis of protein prenylation and prenylation dynamics. Nature Chemistry 11, 552–561. 10.1038/s41557-019-0237-6 PMC6544531 30936521

[erag069-B198] Strasser R . 2018. Protein quality control in the endoplasmic Reticulum of plants. Annual Review of Plant Biology 69, 147–172. 10.1146/annurev-arplant-042817-040331 PMC7039705 29570364

[erag069-B199] Subramaniam G, Trusov Y, Lopez-Encina C, Hayashi S, Batley J, Botella JR. 2016. Type B heterotrimeric G protein *γ*-subunit regulates auxin and ABA signaling in tomato. Plant Physiology 170, 1117–1134. 26668332 10.1104/pp.15.01675 PMC4734580

[erag069-B200] Suzuki M, Kamide Y, Nagata N, et al 2004. Loss of function of *3-hydroxy-3-methylglutaryl coenzyme A reductase 1* (*HMG1*) in *Arabidopsis* leads to dwarfing, early senescence and male sterility, and reduced sterol levels. The Plant Journal 37, 750–761. 14871314 10.1111/j.1365-313x.2004.02003.x

[erag069-B201] Suzuki M, Nakagawa S, Kamide Y, Kobayashi K, Ohyama K, Hashinokuchi H, Kiuchi R, Saito K, Muranaka T, Nagata N. 2009. Complete blockage of the mevalonate pathway results in male gametophyte lethality. Journal of Experimental Botany 60, 2055–2064. 19363204 10.1093/jxb/erp073 PMC2682496

[erag069-B202] Swiezewska E, Dallner G, Andersson B, Ernster L. 1993. Biosynthesis of ubiquinone and plastoquinone in the endoplasmic reticulum-Golgi membranes of spinach leaves. The Journal of Biological Chemistry 268, 1494–1499. 8419349

[erag069-B203] Tateishi M, Goto K, Hishinuma E, Matsukawa N, Kishimoto T, Tanaka K, Horiuchi H, Fukasawa M, Shirakawa R. 2025. Double prenylation of budding yeast Ykt6 regulates cell wall integrity and autophagy. The Journal of Biological Chemistry 301, 108384. 40049413 10.1016/j.jbc.2025.108384 PMC12001115

[erag069-B204] Tejos RI, Mercado AV, Meisel LA. 2010. Analysis of chlorophyll fluorescence reveals stage specific patterns of chloroplast-containing cells during Arabidopsis embryogenesis. Biological Research 43, 99–111. 21157637

[erag069-B205] Terceros GC, Resentini F, Cucinotta M, Manrique S, Colombo L, Mendes MA. 2020. The importance of cytokinins during reproductive development in Arabidopsis and beyond. International Journal of Molecular Sciences 21, 8161. 33142827 10.3390/ijms21218161 PMC7662338

[erag069-B206] Thai L, Rush JS, Maul JE, Devarenne T, Rodgers DL, Chappell J, Waechter CJ. 1999. Farnesol is utilized for isoprenoid biosynthesis in plant cells via farnesyl pyrophosphate formed by successive monophosphorylation reactions. Proceedings of the National Academy of Sciences, USA 96, 13080–13085. 10.1073/pnas.96.23.13080 PMC23903 10557276

[erag069-B207] Theesfeld CL, Hampton RY. 2013. Insulin-induced gene protein (INSIG)-dependent sterol regulation of Hmg2 endoplasmic reticulum-associated degradation (ERAD) in yeast. The Journal of Biological Chemistry 288, 8519–8530. 23306196 10.1074/jbc.M112.404517 PMC3605666

[erag069-B208] Thole JM, Perroud PF, Quatrano RS, Running MP. 2014. Prenylation is required for polar cell elongation, cell adhesion, and differentiation in *Physcomitrella patens*. The Plant Journal 78, 441–451. 24634995 10.1111/tpj.12484

[erag069-B209] Tholl D . 2006. Terpene synthases and the regulation, diversity and biological roles of terpene metabolism. Current Opinion in Plant Biology 9, 297–304. 16600670 10.1016/j.pbi.2006.03.014

[erag069-B210] Thoma NH, Niculae A, Goody RS, Alexandrov K. 2001. Double prenylation by RabGGTase can proceed without dissociation of the mono-prenylated intermediate. The Journal of Biological Chemistry 276, 48631–48636. 11591706 10.1074/jbc.M106470200

[erag069-B211] Tian C, Wang Q. 2025. Protein prenylation in plants: mechanisms and functional implications. Plants 14, 1759. 40573747 10.3390/plants14121759 PMC12197051

[erag069-B212] Trueblood CE, Ohya Y, Rine J. 1993. Genetic evidence for in vivo cross-specificity of the CaaX-box protein prenyltransferases farnesyltransferase and geranylgeranyltransferase-I in Saccharomyces cerevisiae. Nature Reviews: Molecular Cell Biology 13, 4260–4275. 10.1128/mcb.13.7.4260-4275.1993 PMC359976 8321228

[erag069-B213] Valentin HE, Lincoln K, Moshiri F, et al 2005. The *Arabidopsis vitamin E pathway gene5-1* mutant reveals a critical role for phytol kinase in seed tocopherol biosynthesis. The Plant Cell 18, 212–224. 16361393 10.1105/tpc.105.037077 PMC1323494

[erag069-B214] Varki A . 2017. Biological roles of glycans. Glycobiology 27, 3–49. 27558841 10.1093/glycob/cww086 PMC5884436

[erag069-B215] Verdaguer IB, Crispim M, Hernandez A, Katzin AM. 2022. The biomedical importance of the missing pathway for farnesol and geranylgeraniol salvage. Molecules 27, 8691. 36557825 10.3390/molecules27248691 PMC9782597

[erag069-B216] Verges V, Dutilleul C, Godin B, et al 2021. Protein farnesylation takes part in Arabidopsis seed development. Frontiers in Plant Science 12, 620325. 33584774 10.3389/fpls.2021.620325 PMC7876099

[erag069-B217] Vom Dorp K, Holzl G, Plohmann C, Eisenhut M, Abraham M, Weber AP, Hanson AD, Dormann P. 2015. Remobilization of phytol from chlorophyll degradation is essential for tocopherol synthesis and growth of Arabidopsis. The Plant Cell 27, 2846–2859. 26452599 10.1105/tpc.15.00395 PMC4682318

[erag069-B218] Vranova E, Coman D, Gruissem W. 2013. Network analysis of the MVA and MEP pathways for isoprenoid synthesis. Annual Review of Plant Biology 64, 665–700. 10.1146/annurev-arplant-050312-120116 23451776

[erag069-B219] Vranova E, Kopcsayova D, Kosuth J, Colinas M. 2019. Mutant-based model of two independent pathways for carotenoid-mediated chloroplast biogenesis in Arabidopsis embryos. Frontiers in Plant Science 10, 1034. 31507624 10.3389/fpls.2019.01034 PMC6718698

[erag069-B220] Wang M, Casey PJ. 2016. Protein prenylation: unique fats make their mark on biology. Nature Reviews: Molecular Cell Biology 17, 110–122. 26790532 10.1038/nrm.2015.11

[erag069-B221] Wang Y, Ying J, Kuzma M, et al 2005. Molecular tailoring of farnesylation for plant drought tolerance and yield protection. The Plant Journal 43, 413–424. 16045476 10.1111/j.1365-313X.2005.02463.x

[erag069-B222] Wang Y, Beaith M, Chalifoux M, Ying J, Uchacz T, Sarvas C, Griffiths R, Kuzma M, Wan J, Huang Y. 2009. Shoot-specific down-regulation of protein farnesyltransferase (alpha-subunit) for yield protection against drought in canola. Molecular Plant 2, 191–200. 19529821 10.1093/mp/ssn088 PMC2639732

[erag069-B223] Wang Z, Nelson DR, Zhang J, Wan X, Peters RJ. 2023. Plant (di)terpenoid evolution: from pigments to hormones and beyond. Natural Product Reports 40, 452–469. 36472136 10.1039/d2np00054g PMC9945934

[erag069-B224] Wangeline MA, Hampton RY. 2018. “Mallostery”—ligand-dependent protein misfolding enables physiological regulation by ERAD. The Journal of Biological Chemistry 293, 14937–14950. 30018140 10.1074/jbc.RA118.001808 PMC6153289

[erag069-B225] Wangeline MA, Hampton RY. 2021. An autonomous, but INSIG-modulated, role for the sterol sensing domain in mallostery-regulated ERAD of yeast HMG-CoA reductase. The Journal of Biological Chemistry 296, 100063. 33184059 10.1074/jbc.RA120.015910 PMC7948459

[erag069-B226] Wanke M, Dallner G, Swiezewska E. 2000. Subcellular localization of plastoquinone and ubiquinone synthesis in spinach cells. Biochimica et Biophysica Acta (BBA) - Biomembranes 1463, 188–194. 10631308 10.1016/s0005-2736(99)00191-1

[erag069-B227] Watkins JL . 2023. Uncovering the secrets to vibrant flowers: the role of carotenoid esters and their interaction with plastoglobules in plant pigmentation. New Phytologist 240, 7–9. 37547993 10.1111/nph.19185

[erag069-B228] Xing S, Miao J, Li S, Qin G, Tang S, Li H, Gu H, Qu LJ. 2010. Disruption of the 1-deoxy-D-xylulose-5-phosphate reductoisomerase (DXR) gene results in albino, dwarf and defects in trichome initiation and stomata closure in Arabidopsis. Cell Research 20, 688–700. 20404857 10.1038/cr.2010.54

[erag069-B229] Xu JJ, Hu M, Yang L, Chen XY. 2022. How plants synthesize coenzyme Q. Plant Communications 3, 100341. 35614856 10.1016/j.xplc.2022.100341 PMC9483114

[erag069-B230] Yalovsky S, Rodriguez-Concepcion M, Bracha K, Toledo-Ortiz G, Gruissem W. 2000. Prenylation of the floral transcription factor APETALA1 modulates its function. The Plant Cell 12, 1257–1266. 10948247 10.1105/tpc.12.8.1257 PMC149100

[erag069-B231] You MK, Lee YJ, Yu JS, Ha SH. 2020. The predicted functional compartmentation of rice terpenoid metabolism by trans-prenyltransferase structural analysis, expression and localization. International Journal of Molecular Sciences 21, 8927. 33255547 10.3390/ijms21238927 PMC7728057

[erag069-B232] Zeng Q, Wang X, Running MP. 2007. Dual lipid modification of Arabidopsis G*γ*-subunits is required for efficient plasma membrane targeting. Plant Physiology 143, 1119–1131. 17220359 10.1104/pp.106.093583 PMC1820929

[erag069-B233] Zhang X, Sun S, Nie X, Boutte Y, Grison M, Li P, Kuang S, Men S. 2016. Sterol methyl oxidases affect embryo development via auxin-associated mechanisms. Plant Physiology 171, 468–482. 27006488 10.1104/pp.15.01814 PMC4854682

[erag069-B234] Zhang X, Cao S, Barila G, Edreira MM, Hong K, Wankhede M, Naim N, Buck M, Altschuler DL. 2018. Cyclase-associated protein 1 (CAP1) is a prenyl-binding partner of Rap1 GTPase. The Journal of Biological Chemistry 293, 7659–7673. 29618512 10.1074/jbc.RA118.001779 PMC5961064

[erag069-B235] Zhou Y, Hancock JF. 2018. Electron microscopy combined with spatial analysis: quantitative mapping of the nano-assemblies of plasma membrane-associating proteins and lipids. Biophysics Reports 4, 320–328. 30596140 10.1007/s41048-018-0060-4 PMC6276063

[erag069-B236] Zhou Y, Hancock JF. 2020. A novel prenyl-polybasic domain code determines lipid-binding specificity of the K-ras membrane anchor. Small GTPases 11, 220–224. 29239694 10.1080/21541248.2017.1379583 PMC7549719

[erag069-B237] Zhou Y, Prakash PS, Liang H, Gorfe AA, Hancock JF. 2021. The KRAS and other prenylated polybasic domain membrane anchors recognize phosphatidylserine acyl chain structure. Proceedings of the National Academy of Sciences, USA 118, e2014605118. 10.1073/pnas.2014605118 PMC8017956 33526670

[erag069-B238] Zhou Y, Dobritsa AA. 2025. Forging the pollen fortress: cell biological mechanisms of exine formation. Current Opinion in Plant Biology 86, 102742. 40482301 10.1016/j.pbi.2025.102742

[erag069-B239] Zi J, Mafu S, Peters RJ. 2014. To gibberellins and beyond! surveying the evolution of (di)terpenoid metabolism. Annual Review of Plant Biology 65, 259–286. 10.1146/annurev-arplant-050213-035705 PMC4118669 24471837

[erag069-B240] Ziegelhoffer EC, Medrano LJ, Meyerowitz EM. 2000. Cloning of the *Arabidopsis WIGGUM* gene identifies a role for farnesylation in meristem development. Proceedings of the National Academy of Sciences, USA 97, 7633–7638. 10.1073/pnas.130189397 PMC16597 10840062

